# Single-cell transcriptomic profiling of the mouse cochlea: An atlas for targeted therapies

**DOI:** 10.1073/pnas.2221744120

**Published:** 2023-06-20

**Authors:** Philippe Jean, Fabienne Wong Jun Tai, Amrit Singh-Estivalet, Andrea Lelli, Cyril Scandola, Sébastien Megharba, Sandrine Schmutz, Solène Roux, Sabrina Mechaussier, Muriel Sudres, Enguerran Mouly, Anne-Valérie Heritier, Crystel Bonnet, Adeline Mallet, Sophie Novault, Valentina Libri, Christine Petit, Nicolas Michalski

**Affiliations:** ^a^Institut Pasteur, Université Paris Cité, INSERM, Institut de l’Audition, Plasticity of Central Auditory Circuits, F-75012 Paris, France; ^b^Institut Pasteur, Université Paris Cité, INSERM, Institut de l’Audition, Auditory Therapies Innovation Laboratory, F-75012 Paris, France; ^c^Institut Pasteur, Université Paris Cité, Ultrastructural BioImaging, F-75015 Paris, France; ^d^Institut Pasteur, Université Paris Cité, Cytometry and Biomarkers, F-75015 Paris, France; ^e^Collège de France, F-75005 Paris, France

**Keywords:** hearing, gene therapy, cochlea, tonotopy, transcriptomics

## Abstract

Functional molecular characterization of the cochlea has mainly been driven by the deciphering of the genetic architecture of sensorineural deafness. As a result, the search for curative treatments, which are sorely lacking in the hearing field, has become a potentially achievable objective, particularly *via* cochlear gene and cell therapies. To this end, a complete inventory of cochlear cell types, with an in-depth characterization of their gene expression profiles right up to their final differentiation, is indispensable. We therefore generated a single-cell transcriptomic atlas of the mouse cochlea based on an analysis of more than 120,000 cells on postnatal day 8 (P8), during the prehearing period, P12, corresponding to hearing onset, and P20, when cochlear maturation is almost complete. By combining whole-cell and nuclear transcript analyses with extensive in situ RNA hybridization assays, we characterized the transcriptomic signatures covering nearly all cochlear cell types and developed cell type–specific markers. Three cell types were discovered; two of them contribute to the modiolus which houses the primary auditory neurons and blood vessels, and the third one consists in cells lining the scala vestibuli. The results also shed light on the molecular basis of the tonotopic gradient of the biophysical characteristics of the basilar membrane that critically underlies cochlear passive sound frequency analysis. Finally, overlooked expression of deafness genes in several cochlear cell types was also unveiled. This atlas paves the way for the deciphering of the gene regulatory networks controlling cochlear cell differentiation and maturation, essential for the development of effective targeted treatments.

## Significance

An increasing number of therapeutic strategies are being designed and tested in animal models for numerous forms of hereditary deafness, the most frequent genetic sensorineural disorder. One major challenge is the implementation of these therapies for diverse isolated and syndromic forms of hearing loss, taking into account the spatial and temporal patterns of expression of the causal gene in the auditory sensory organ, the cochlea. Here, combining single-cell and single-nucleus RNA sequencing with in situ RNA hybridization assays, we present a large-scale single-cell transcriptomic atlas for three crucial stages in the maturation of the mouse cochlea. This detailed atlas of gene expression provides key information for the development of effective therapeutic approaches.

The auditory system is unique among mammalian sensory systems in terms of its extreme biophysical performance. The paucity of cochlear cells, especially the sensory hair cells (HCs)—fewer than 15,000 in a human cochlea—long hampered efforts to decipher the molecular mechanisms underlying the development and physiology of this organ. Moreover, the spatial frequency organization of the cochlea, the tonotopy, adds to the heterogeneity of most cochlear cell types, as the morphology and physiological properties of any given cell type vary gradually along the tonotopic axis, from the base to the apex of the cochlea. Within the sensory epithelium, the organ of Corti, housing the HCs and their supporting cells (SCs), lies on the basilar membrane, which is also endowed with tonotopically ordered biophysical characteristics. Hereditary deafness, the most frequent disabling sensorineural disorder, is estimated to account for 60 to 80% of congenital or permanent prelingual forms of deafness in high-income countries (1). Congenital and prelingual inherited forms, and those with an onset up to early adulthood, are almost exclusively monogenic and display considerable genetic heterogeneity, with about 125 genes responsible for isolated (nonsyndromic) and about 300 genes responsible for syndromic forms identified to date (2). Some of the causal genes for inherited forms of deafness play a crucial role in one particular cochlear cell type, as exemplified by most of the genes encoding the molecular machinery for mechanoelectrical transduction in HCs, which is located in the hair bundle, the mechanoreceptive structure for sound stimulation. Other genes play crucial roles in several cochlear cell types. For example, *GJB2*, encoding connexin 26, is expressed in most cochlear cell types other than HCs. Gene therapy strategies are increasingly being designed and tested in mouse models of human deafness, with the aim of rescuing hearing by gene replacement, augmentation, or editing in the cochlear cell type(s) directly affected by the deficit. Proofs of concept for positive effects have already been established in mouse models for several human deafness forms (3–6). However, the optimization of these procedures to ensure efficient and safe therapies with durable effects is critically dependent on adequate levels of temporal and spatial expression for the therapeutic agent. For this purpose, the building of a single-cell transcriptomic reference atlas for the cochlea is an indispensable step.

The application of recent advances in microfluidics to the sorting of isolated cells extended single-cell deep-sequencing approaches to large numbers of cells, thereby increasing the transcriptomic information that can be harvested from a tissue by several orders of magnitude. Some in-depth transcriptomic studies have already investigated specific cochlear cell types, such as the stria vascularis cells (7, 8), some SC types (9), and the primary sensory neurons of the auditory nerve (10–13). By contrast, larger studies at the tissue scale were initiated much more recently, with some being available to the scientific community (14). Among them, single-cell transcriptomic experiments have been performed on cochlear floor epithelia obtained during embryogenesis and up to P7 in mice, thus characterizing the developmental trajectories of cochlear cell types and their early differentiation steps (15). Nevertheless, there is currently no comprehensive standardized transcriptomic atlas for all cochlear cell types covering the entire differentiation process of this sensory organ right up to full maturation. In the case of genetic defects causing particular pathophysiological conditions, we need to assess not only the transcriptomic changes of the target cell types but also of the neighboring ones at various time points if we are to detect possible indirect impacts on these cells. A reference atlas is also required for a full comprehension of aging as well as the effects of environmental insults, such as ototoxic drug exposure (16) or noise, on the various cell types of the cochlea, the characterizations of which have only just begun (17, 18).

Given that large exhaustive transcriptomic datasets for time points after P7 are currently lacking in mice, we here built a comprehensive transcriptomic atlas of the mouse cochlea based on the analysis of more than 120,000 cells at three critical stages of differentiation: P8 (before hearing onset), P12 (upon hearing onset), and P20 (when the cochlea is mostly mature). We obtained transcriptomic signatures for almost all cochlear cell types by combining single-cell and single-nucleus RNA sequencing (scRNAseq and snRNAseq, respectively) with extensive in situ RNA hybridization assays (RNAscope). Furthermore, we identified three previously unknown cell types and laid the foundations for deciphering the molecular mechanisms underlying establishment of the tonotopic organization of the biophysical properties of the basilar membrane.

## Results

### Adapting scRNAseq and snRNAseq to Capture the Cell Type Diversity in the Cochlea.

**Fig. 1.**
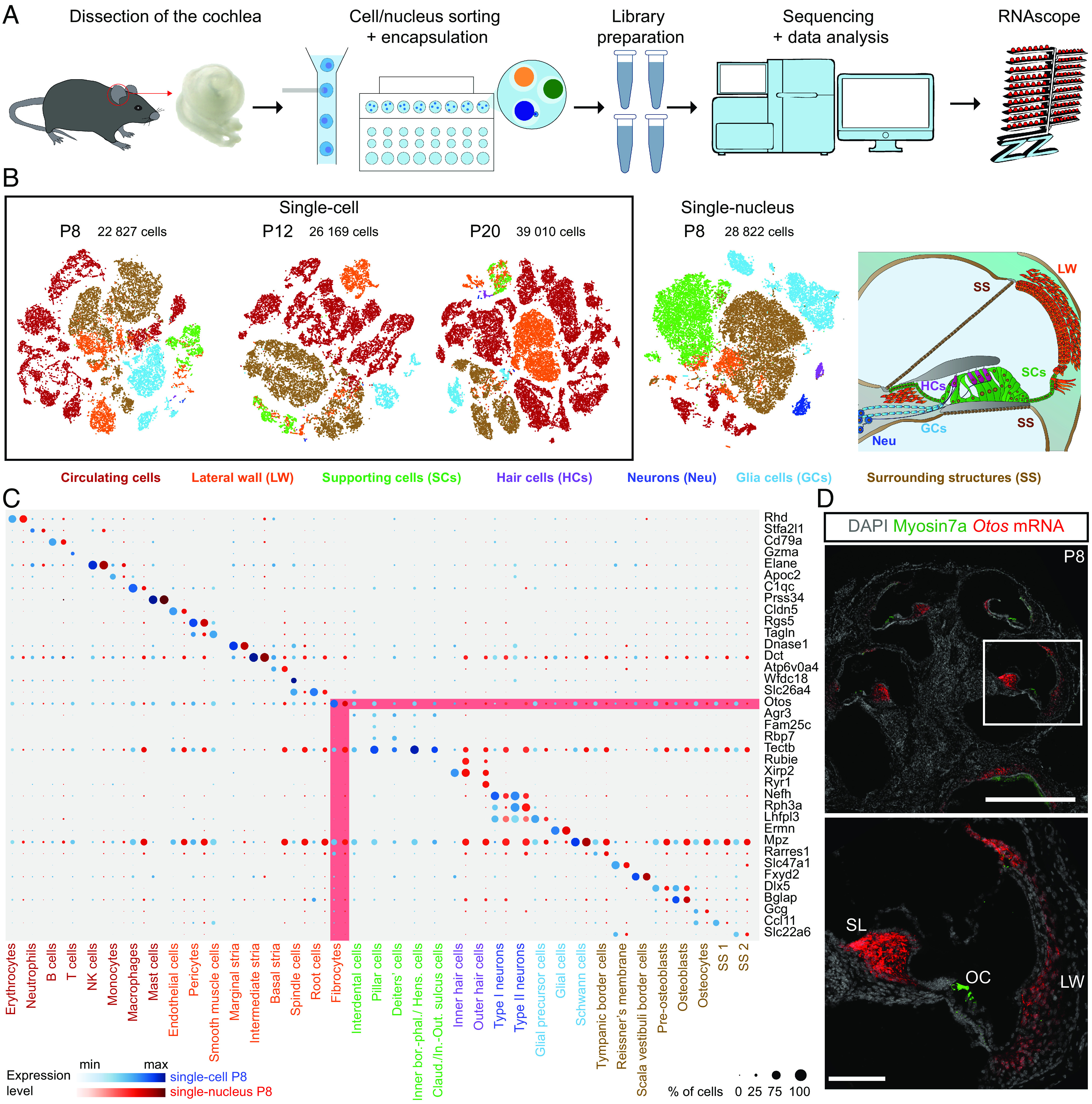
Transcriptomic characterization of cochlear cell types. (*A*) Experimental design of the study. (*B*, *Left*) T-SNE plots depicting the scRNAseq (P8-P12-P20) and the snRNAseq (P8) datasets. (*B*, *Right*) Diagram of the cochlea depicting the main cell-type ensembles characterized. (*C*) Bubble plot analysis of the most differentially expressed gene for each cochlear cell type, comparing the scRNAseq and snRNAseq datasets on P8. The expression of *Otos* is highlighted in red. (*D*) Top window (scale bar, 500 µm): Z-projection of a P8 whole cochlea cryosection stained with DAPI, immunostained for myosin7a, and stained for *Otos* mRNA with RNAscope. Bottom window (scale bar, 100 µm): Magnification of the inset showing *Otos* mRNA detection in the spiral limbus (SL) and lateral wall (LW). OC: organ of Corti.

With the aim of building a comprehensive transcriptomic atlas of the mouse cochlea capturing all cell types, we first optimized and standardized the preparation of single cells and nuclei to improve yields for the three postnatal ages tested (P8, P12, and P20). By applying the 10× Genomics technique for scRNAseq and snRNAseq, a total of 22,827, 26,169, and 39,010 cells were obtained from P8, P12, and P20 cochlear samples, respectively, and 28,822 nuclei from P8 cochlear samples (Fig. 1 *A* and *B*, *SI Appendix*, Figs. S1 and S2, and *Materials and Methods*). Based on the analysis of the most differentially expressed genes between cell types (see Dataset S1 and *Material and Methods*) as well as known markers, a total of 37 cell types were identified in the transcriptomic atlas, as shown by the snapshot comparing scRNAseq and snRNAseq data (Fig. 1*C*). In order to confirm cell type assignment, RNAscope assays targeting the most differentially expressed transcripts were used. This is illustrated by probes directed against the *Otos* mRNA that confirmed the localization of *Otos* transcripts (specific of the cochlear fibrocytes) encoding otospiralin, which are present in fibrocytes of the spiral ligament and spiral limbus (Fig. 1*D*) (19).

### Broad Characterization of Cochlear Cell Type Diversity.

We combined the single-cell transcriptomic data from the three differentiation stages probed, corresponding to a total of 88,006 cells, and split the dataset into two main ensembles: circulating cells, mostly blood cells, and noncirculating cells (resident cochlear cells) that were further split into cells from soft and cartilaginous/bony cochlear tissues. The circulating cells were manually characterized on the basis of canonical marker expression (*SI Appendix*, Fig. S3 *A*–*C*). Noncirculating cell types were subdivided into six main ensembles according to their cochlear subregion of origin, namely the cell ensemble of the lateral wall that is mainly represented by the stria vascularis cells in our scRNAseq data, especially with its intermediate layer derived of neural crest (identified by *Mlana* coding for a melanoma antigen); the cell ensembles of the neurosensory epithelium, which consists of SCs (identified by *Otog* encoding otogelin, *SI Appendix*, Fig. S5*B*) and HCs (by *Capb2* encoding the calcium-binding protein 2); as well as the cell ensemble of the spiral ganglion, which is composed of neurons (identified by *Nefh* coding for neurofilament H) and glial cells (identified by *Gjb1* coding for connexin 32). Finally, the remaining cochlear cell types were grouped together under the cell ensemble designated as “surrounding structures” (*SI Appendix*, Fig. S3 *D*–*F*); they express the *Cavin2* gene encoding caveolae-associated protein 2 and their analysis led to the uncovering of cell types. For the sake of clarity, the atlas resulting from the identification of all the cell types in the dataset is presented using this subdivision.

### Transcriptomic Profiles of Individual Cochlear Lateral Wall Cell Types and Associated Cell Markers.

**Fig. 2.**
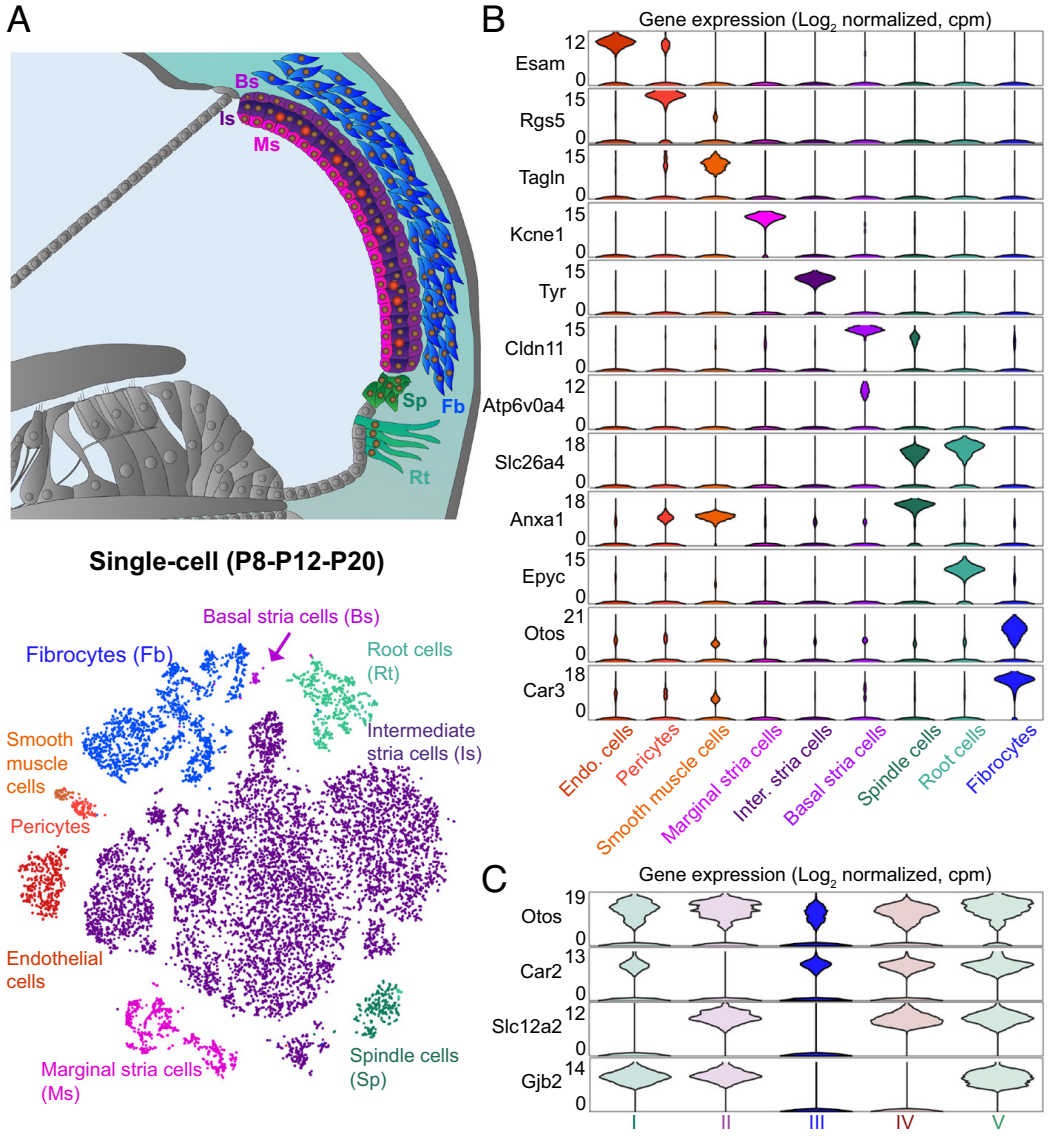
Characterization of lateral wall cell types and identification of markers. (*A*, *Top*) Diagram illustrating the lateral wall of the cochlea. The transcriptomic data identified the root (Rt) and spindle (Sp) cells; fibrocytes (Fb); and the stria vascularis composed of the marginal (Ms), intermediate (Is), and basal stria (Bs) cells. (*A*, *Bottom*) t-SNE plot of the scRNAseq (P8-P12-P20) datasets for the lateral wall cells. (*B*) Violin plots showing expression levels for a subset of the genes used to classify the cell types. (*C*) Classification of fibrocytes into five subtypes based on the differential expression of several genes.

The transepithelial potential between the endolymph and the perilymph drives transduction current through the mechanosensitive channels in the hair bundle of the HCs. By establishing and maintaining the endocochlear potential, the lateral wall, composed of the stria vascularis and the spiral ligament (20) (Fig. 2*A*), plays a key role in auditory function. Blood capillaries could be identified through the transcriptomic signature of the endothelial cells based on endothelial cell adhesion molecular transcript (encoded by *Esam*) expression. Pericytes could be identified based on the regulator of G-protein signaling 5 transcript (encoded by *Rgs5*) expression (21), while smooth muscle cells were identified based on transgelin transcript (encoded by *Tagln*) expression (22) (Fig. 2*B*). For the sake of simplicity in data presentation, all the capillaries of our dataset were assigned, by default, to the stria vascularis housing 80% of the cochlear capillaries (23).

We could identify the cell types composing the three layers of the stria vascularis, the main interface for exchanges between the lateral wall and the endolymphatic compartment. The cells of the outer layer, the marginal cells, were identified on the basis of the specific expression of the K^+^ channel subunit *Kcne1* (Fig. 2*B*), and the Cl^−^ channel subunit Barttin (*Bsnd*) (*SI Appendix*, Figs. S10–13) (24, 25). The cells of the intermediate layer, which have the same embryonic origin as melanocytes, could be identified on the basis of the expression of genes involved in melanin biosynthesis (26), such as *Tyr* encoding tyrosinase (Fig. 2*B*), *Dct* encoding dopachrome tautomerase, and *Slc45a2* encoding a membrane-associated proton/glucose transporter protein. The innermost layer, made of basal stria cells, lacks of specific markers. *Cldn11*, the most commonly used one, is strongly expressed in basal stria cells (27) (Fig. 2*B*), but is also present in other cochlear cell types (*SI Appendix*, Fig. S4 *A* and *B*). Based on the expression of this gene, we identified a candidate cluster of this cell type. In this cell population, the most differentially expressed gene encoded the ATPase proton pump Atp6v0a4 (Fig. 2*B*). Atp6v0a4 is defective in distal renal tubular acidosis in humans, a condition often associated with sensorineural hearing loss (28), and defects in *Atp6v0a4* have been shown to cause severe hearing impairment in mice. *Atp6v0a4* expression has been previously described in the endolymphatic sac, a nonsensory organ of the inner ear. However, RNAscope assays revealed an expression of *Atp6v0a4* transcripts exclusively in the basal stria layer, providing a specific marker of the basal stria cells (*SI Appendix*, Fig. S4*B*). This observation suggests that these cells are likely involved in the pathogenesis of the hearing impairment in distal renal tubular acidosis.

We then identified the spindle cells and root cells located between the stria vascularis and the outer sulcus cells, based on the expression of *Slc26a4*, encoding the anion exchange protein, pendrin (Fig. 2*B*), whose homozygous mutations cause the deafness-associated Pendred syndrome (29, 30). The functions of these cells remain unclear, but a possible role in cochlear K^+^ homeostasis has been suggested (24). These two cell types were discriminated on the basis of the differential expression of epiphycan (*Epyc*) and annexin A1 (*Anxa1*) expressed in root cells and spindle cells, respectively (Fig. 2*B*) (7). We then identified the fibrocytes, the main cellular component of the spiral ligament, a structure known for its elasticity that enables the displacement of the basilar membrane. These cells form the loose connective tissues and are crucial for maintenance of the endocochlear potential and blood flow regulation (19). They were identified on the basis of their expression of a specific marker, otospiralin, and expressed specific collagen transcripts such as *Col9a2* and *Col9a3* (*SI Appendix*, Fig. S4*C*). Fibrocytes were further subdivided into their five known subtypes present in the spiral ligament by using the *Slc12a2*, *Gjb2,* and *Car2* markers, which led us to generate their respective transcriptomic profile (Fig. 2*C* and Dataset S1). In addition to the spiral ligament, fibrocytes are also present in the spiral limbus (*SI Appendix*, Fig. S4*C*), a region localized medially to the neurosensory epithelium. Taking advantage of this atlas, further RNAscope assays will be critical to know whether the spiral limbus expresses those subtypes, or additional ones. Notably, the snRNAseq method brought more balanced cell population sizes, an observation particularly striking for the three stria vascularis layers (*SI Appendix*, Fig. S4*D*). Indeed, the scRNAseq method resulted in a strong positive bias toward the harvesting of the intermediate stria cells at the expense of other cell types, especially the basal stria cells that were almost excluded. Overall, through the unprecedented characterization of the five subtypes of fibrocytes and the identification of associated cell markers, our atlas provides a deep transcriptomic profiling of all lateral wall cell types and opens avenues for the developmental analysis of their transcriptome.

### Refined Signatures of Supporting Cell Types of the Neurosensory Epithelium.

**Fig. 3.**
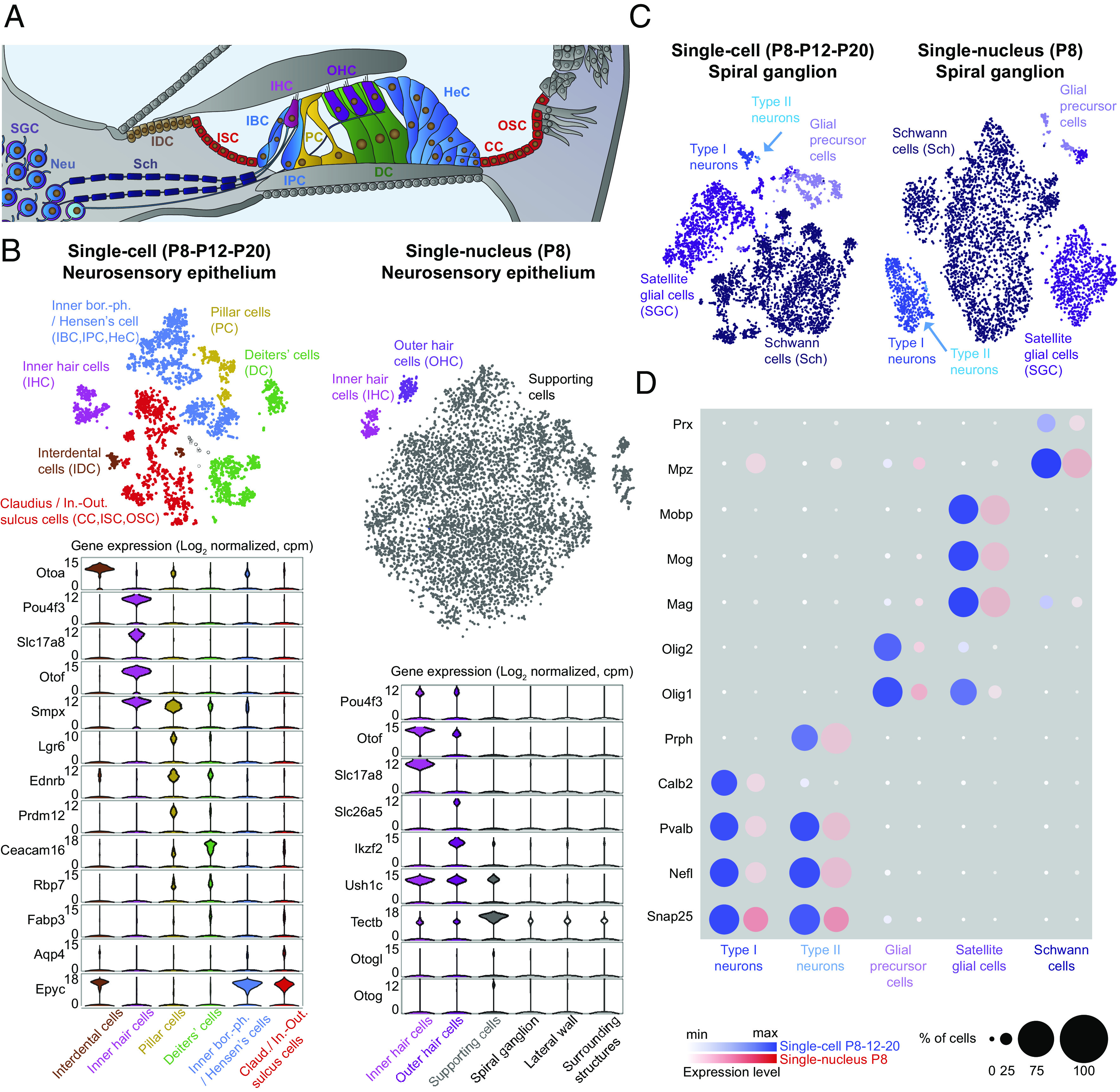
Transcriptomic classification of the neurosensory epithelium and spiral ganglion. (*A*) Diagram of the cochlea, highlighting the neurosensory epithelium and afferent connections from the ganglion. The transcriptomic data identified one group of cells comprising the inner border (IBC), inner phalangeal (IPC), and Hensen’s (HeC) cells; another group of cells comprising the inner–outer sulcus (ISC-OSC) and Claudius cells (CC); and individual cell types, such as the interdental (IDC), pillar (PC), and Deiter’s cells (DC). The type I and II neurons were characterized together with the satellite glial cells (SGCs) and Schwann cells (Sch). (*B*) ScRNAseq (P8-P12-P20) (*Left*) and P8 snRNAseq (*Right*) datasets for the neurosensory epithelium. (*Top*) Corresponding t-SNE plots. The black circles correspond to nonassigned cells. (*Bottom*) Corresponding violin plots showing expression levels for a subset of genes used to classify cell types. (*C*) t-SNE plots for scRNAseq (P8-P12-P20) and snRNAseq (P8) on neuronal/glial cells. (*D*) Bubble plot analysis comparing the scRNAseq (P8-P12-P20) and snRNAseq (P8) datasets.

When undertaking the classification of sensory cells in our scRNAseq data, inner hair cells (IHCs) were easily identified on the basis of the expression of *Pou4f3,* encoding a HC transcription factor (31) expressed by both IHCs and outer hair cells (OHCs), and the genes encoding the glutamate transporter Vglut3 (*Slc17a8*) as well as the calcium sensor of synaptic vesicle recycling and exocytosis otoferlin (*Otof * ), both of which are specific to IHCs on P8 (3, 32). In the absence of detection of *Slc26a5,* encoding the piezoelectric protein prestin, which mediates OHC electromotility and is involved in cochlear amplification (33), as well as *Ikzf2,* a key transcriptional regulator involved in OHC maturation (34), we concluded to the absence of OHCs from our scRNAseq data. Likely, they were excluded during the cell sorting steps, due to their fragility and atypical membrane properties related to the presence of prestin. In contrast, the snRNAseq dataset enabled to detect both IHCs and OHCs as two separate clusters, using the markers previously mentioned for their identification (Fig. 3 *A* and *B*).

We then tackled the identification of the various epithelial SCs that are interconnected by tight and adherens junctions maintaining the structural integrity of the epithelium and its resilience to sound stimulation, and that communicate by gap junctions. SCs ensure the impermeability of the barrier between the endolymph and the perilymph, and thereby the endocochlear potential essential for HC transduction. The characterization of the various SC types by their transcriptomic profiling is hampered by the absence of known specific markers allowing to distinguish one SC type to another. Combining scRNAseq data with RNAscope assays, the SCs were identified on the basis of the presence of transcripts encoding for otogelin (*Otog*) and otogelin-like (*Otogl*), which are known to form the horizontal top connectors and the tectorial membrane-attachment crowns of the OHC hair bundles (35). This identification was further confirmed by the presence of transcripts encoding the tectorial membrane-like tectorin-β (*Tectb*) and harmonin (*Ush1c*) proteins, the latter being in addition expressed in HCs as previously described (Fig. 3 *A* and *B* and *SI Appendix*, Fig. S5 *A* and *B*) (36). By performing RNAscope assays and on the basis of published data (37, 38), we then grouped some SC types into two main groups according to the differential expression of *Smp*x, encoding the small muscle protein X, and *Epyc* encoding an extracellular matrix component, epiphycan. The inner border, inner phalangeal, and Hensen’s cells express both *Smpx* and *Epyc,* whereas the Claudius cells and inner and outer sulcus cells express *Epyc* only. Aquaporin 4 by RNAscope assay was found to have an expression profile similar to that of *Epyc,* agreeing with previously published results (39) and reinforcing the validity of this subdivision (*SI Appendix*, Fig. S5 *C* and *D*). For three additional SC types, it was possible to define their individual transcriptomic signature: the interdental cells, which are located on the inner side of the neurosensory epithelium, and specifically express *Otoa,* encoding otoancorin, a protein essential for attachment of the tectorial membrane to the underneath spiral limbus (40); the pillar cells, which form the tunnel of Corti and were identified on the basis of their strong expression of *Lgr6*, *Ednrb,* and *Prdm12* (41); and the Deiters’ cells which provide a structural scaffold for the OHCs and have been shown to express a number of genes, such as *Ceacam16*, *Rbp*, and *Fabp3,* differentially with respect to other SCs (42) (Fig. 3*B*). Our atlas thus extends the transcriptomic profiles of SCs during cochlear maturation, for which the existing data were scarce.

### Identification of Neuronal and Glial Cells.

We complemented this atlas with the spiral ganglion (Fig. 3 *C* and *D*), which contains the cell bodies of the primary auditory neurons. These afferent neurons are either of type I, which accounts for 95% of the neuronal population, with the synapsing of each bipolar neuron to only one IHC, or of type II, with each neuron connecting onto several OHCs. Type I afferent neurons transmit the sound signal from the IHCs to the cochlear nucleus in the brainstem, whereas type II neurons are thought to be involved in nociception, mediating the sensation of painfully loud sounds (43). We identified afferent neurons on the basis of their expression of known neuronal markers (the SNARE protein Snap25 (*Snap25*), the intermediate filament protein neurofilament L (*Nefl*), and the calcium buffers parvalbumin (*Pvalb*) and calbindin (*Calb*)). We distinguished type I and II neurons on the basis of the specific expression of the intermediate filament protein peripherin (*Prph*) present in the latter (44). We also detected the glial cells, which comprise the satellite glial cells, glial precursor cells, and Schwann cells. The satellite glial cells, that express unique markers different from those expressed in the dorsal root ganglia (45), encapsulate the soma of spiral ganglion neurons and most likely maintain their excitability (46). They express genes encoding proteins involved in myelination, such as the myelin oligodendrocyte glycoprotein (*Mog*), myelin-associated glycoprotein (*Mag*), and myelin-associated oligodendrocyte basic protein (*Mobp*). The glial precursor cells did not express the myelin-related genes, but did express the oligodendrocyte transcription factors *Olig1* and *Olig2*. Finally, our data identified the Schwann cells, which produce the myelin sheath insulating the peripheral neurites of the type I neurons. The absence of Schwann cells has been shown to impair the proper patterning of afferent projections (47).These cells specifically express myelin protein zero (*Mpz*), a major component of the peripheral myelin sheath, and periaxin (*Prx*), a protein involved in its maintenance. The P8 snRNAseq data provided similar results in terms of the proportions of the various cell types and their transcriptomic signatures, but with more neurons harvested (Fig. 3 *C* and *D*). Overall, our atlas profiles the transcriptomic signatures of both neurons and all types of glial cells in the mouse cochlea.

### Identification of Cochlear Osseous Cell Types.

**Fig. 4.**
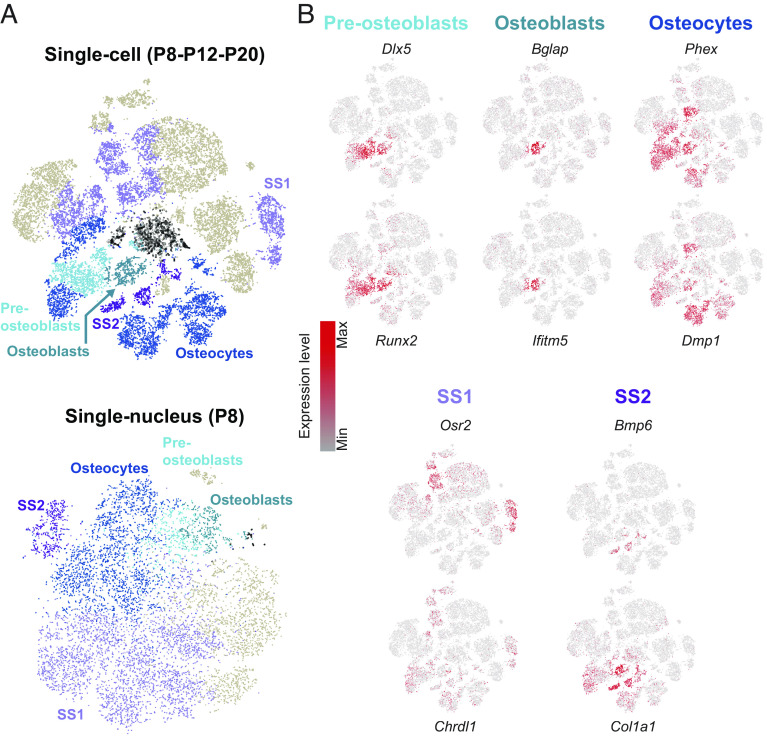
Transcriptomic characterization of the osseous cell types. (*A*) t-SNE plot of scRNAseq (P8-P12-P20) (*Top*) and snRNAseq (P8) (*Bottom*) datasets for the surrounding structures with the preosteoblasts, osteoblasts, osteocytes, and two unknown cell types referred to as surrounding structures 1 (SS1) and 2 (SS2). The black circles correspond to nonassigned cells. (*B*) t-SNE plots of scRNAseq data (P8-P12-P20) showing the most differentially expressed genes relating to bone function.

The remaining noncirculating cells that did not belong to any of the cell-type ensembles described above were classified as surrounding structures. We first classified cochlear cells that expressed genes relating to bone function (Fig. 4). The central axis of the cochlea, the modiolus, is a spongy bone structure from which the osseous spiral lamina is projected about halfway across the cochlear canal. Moreover, the cochlea is surrounded by the osseous shell that was removed during the dissection, but some pieces remained associated with the cochlear tissue, particularly at mature stages (*SI Appendix*, Fig. S1*A*). We identified the three distinct maturation stages of the cells comprising the bone (Fig. 4 *A* and *B*). Cochlear preosteoblasts were identified by analyzing the expression of *Dlx5* and *Runx2* involved in osteoblastic differentiation. They correspond to the first stage of bone cell development from mesenchymal cells and were barely detected from P12 on (*SI Appendix*, Fig. S6*A*). Preosteoblasts develop into osteoblasts, which produce the bone extracellular matrix and its mineralization. Osteoblasts are located over the surface of the bone (*SI Appendix*, Fig. S6*B*), are known to account for 5% of the total bone cell population, and were recognized on the basis of their specific expression of osteocalcin transcript (promoting bone mineralization, encoded by *Bglap*), the interferon-induced transmembrane protein 5 transcript (encoded by *Ifitm5*) (Fig. 4*B*), and the integrin-binding sialoprotein transcript (encoded by *Ibsp*) (*SI Appendix*, Fig. S6*B*). Upon maturation, these cells develop into osteocytes, located deeper in the mineralized bone (*SI Appendix*, Fig. S6*B*), and are known to account for 90 to 95% of the total bone cell population. Markers of osteocytes include the transmembrane endopeptidase *Phex* and the Dentin Matrix Protein 1 *Dmp1* (Fig. 4*B* and *SI Appendix*, Fig. S6*B*) (48).

In addition to these three well-described bone cell types, scRNAseq and snRNAseq also identified two clusters corresponding to two unknown cell types, referred to hereafter as surrounding structures 1 and 2 (SS1, SS2) cells. SS1 differentially expressed genes such as *Osr2* (coding for the Odd-Skipped Related transcription factor 2 protein) and *Chrdl1* (coding for the chordin-like protein 1), whereas SS2 had a different transcriptomic profile, expressing other marker genes, such as *Bmp6* and *Col1a1* at the three tested differentiation stages (Fig. 4*B*). The transcripts of *Aldh1a2*, one of the most differentially expressed genes in SS1 but also expressed in SS2, encoding the Aldehyde Dehydrogenase 1 Family Member A2 enzyme, catalyzing the synthesis of retinoic acid, were detected by RNAscope in the modiolus, the marginal stria cells, and the tympanic border cells (TBCs). The transcripts of *Slc7a11*, one of the most differentially expressed genes in SS2 and absent in SS1, coding for a cystine/glutamate transporter, were also detected in the modiolus but were barely detectable in the other cell types (*SI Appendix*, Fig. S6 *B* and *C*). We then compared the biological functions of SS1 and SS2 with those of all the other cochlear cell types by performing gene ontology (GO) term analysis on their most differentially expressed genes (Dataset S2) that identified biological processes involved in osteoblast differentiation, ossification, cartilage development, odontogenesis, and skeletal system development (*SI Appendix*, Fig. S6*D* and Dataset S2). SS1 and SS2 may, therefore, contribute to bony structures in the modiolus. Whether SS1 and SS2 correspond to two stages of differentiation of the same cell type could not be formally excluded. Altogether, besides the characterization of the three classical bone cells, our data revealed two transcriptomically distinct cell types that we suggest may be related to the spongious nature of the modiolar bone.

### Uncovery and Characterization of a Cochlear Cell Type.

**Fig. 5.**
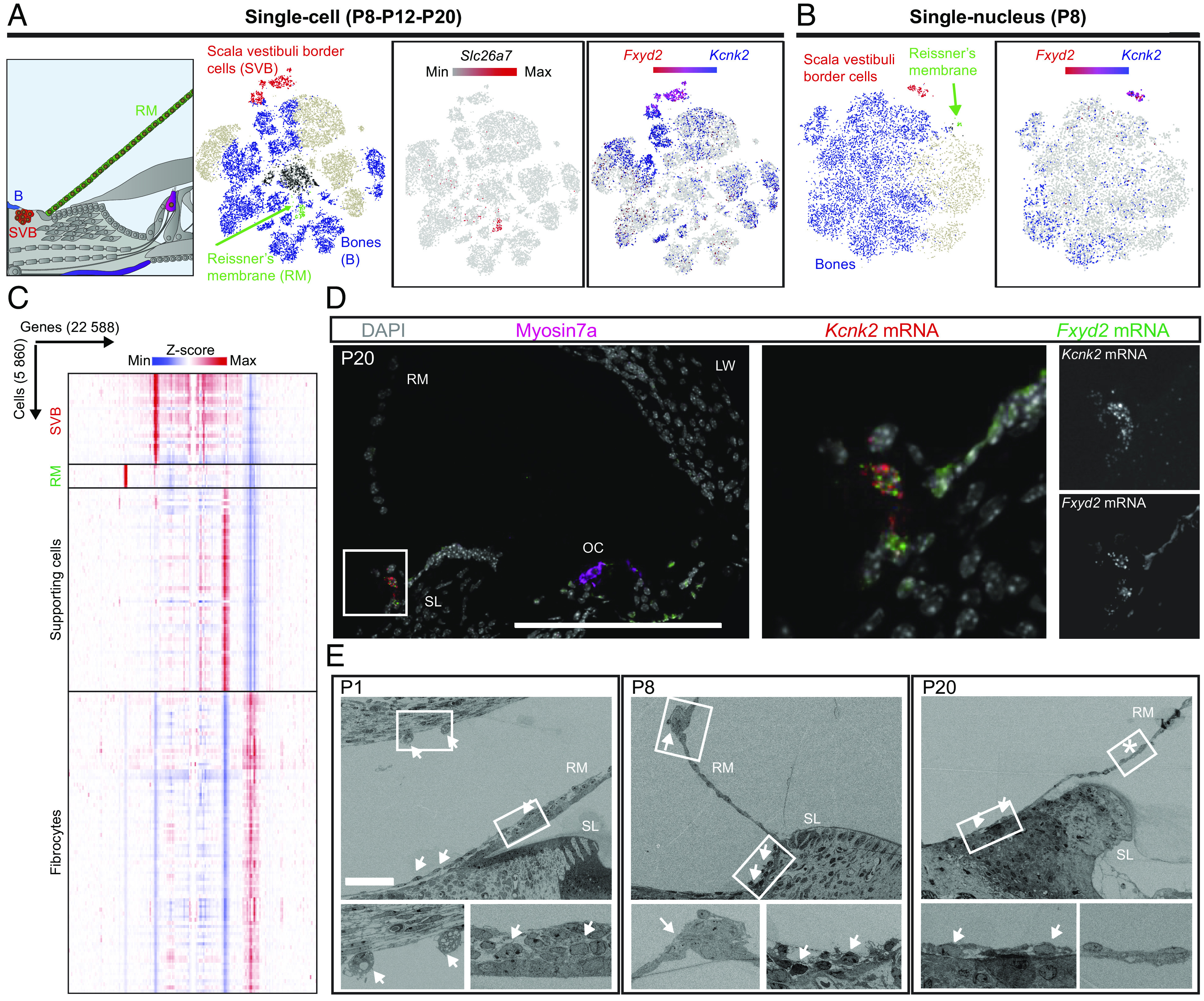
Uncovery of a cochlear cell type. (*A*) Diagram of the cochlea, highlighting Reissner’s membrane (RM) cells, the scala vestibuli border (SVB) cells, and the bone-related cells. *Left* t-SNE plot: scRNAseq (P8-P12-P20) data showing the cell types shown in the diagram. The black circles correspond to nonassigned cells. *Middle* t-SNE plot: scRNAseq (P8-P12-P20) data showing the expression of *Slc26a7*, a marker of RM cells. *Right* t-SNE plot: scRNAseq (P8-P12-P20) datasets showing the expression of *Fxyd2* and *Kcnk2* colocalizing in the SVB cell cluster. (*B*)  *Left* t-SNE plot: snRNAseq P8 data showing the cell types shown in the diagram. *Right* t-SNE plot: snRNAseq P8 data showing the expression of *Fxyd2* and *Kcnk2* colocalizing in the SVB cell cluster. (*C*) Heat map/hierarchical clustering of the SVB cells, RM cells, SCs, and fibrocytes. Gene expression levels are presented as *Z*-scores. (*D*) Z-projections of a P20 cochlea cryosection stained with DAPI, immunostained for myosin7a, and stained for the *Kcnk2* mRNA and *Fxyd2* mRNA with RNAscope. Magnifications of the area framed in white, with individual channels shown. (Scale bar, 100 µm.) (*E*) Serial electron microscopy acquisitions of P1, P8, and P20 cochleae. Magnifications of the areas framed in white are shown. Arrows and asterisk indicate the presence and absence of SVB cells, respectively. (Scale bar, 50 µm.) SL: spiral limbus, RM: Reissner’s membrane, LW: lateral wall, OC: organ of Corti.

In the surrounding structures, we identified three clusters that were not related to the bone. The first one differentially expressed *Slc26a7,* encoding an anion exchange transporter, previously shown by immunostaining to be present in the epithelial cells of Reissner’s membrane (RM) (49). This membrane forms a selective diffusion barrier separating the scala media endolymphatic compartment from the scala vestibuli perilymphatic compartment (Fig. 5*A*) and is therefore an essential structure for the maintenance of a positive endocochlear potential. RNAscope assays against *Slc26a7* mRNA revealed a strong staining of the RM cells at the three stages studied, and a faint staining of the cochlear lateral wall on P8 that had disappeared by P20 (*SI Appendix*, Fig. S7*A*). As *Slc26a7* expression in the lateral wall was barely detectable in the transcriptomic data, this transcript was validated as a specific marker of the RM cells.

The second cluster of cells from nonosseus surrounding structures differentially coexpressed the genes encoding for the Na/K transporting ATPase subunit gamma Fxyd2 and the potassium ion channel Kcnk2, both detected in scRNAseq and snRNAseq data (Fig. 5 *A* and *B*). However, this cluster could not be assigned to any known group of cells. These cells have a transcriptomic profile different from those of RM cells, SCs, fibrocytes (Fig. 5*C*), and all other cochlear cell types. RNAscope assays for the *Fxyd2* and *Kcnk2* transcripts on P12 and P20 localized them in a cluster of cells medial to the neurosensory epithelium, a few micrometers away from the base of RM and facing the scala vestibuli at every turn of the cochlea (Fig. 5*D* and *SI Appendix*, Fig. S7*A*). Earlier, on P8, the cell cluster extended further, reaching the base of RM (Fig. 5*E* and *SI Appendix*, Fig. S7*A*). At earlier stages, on E16.5, the *Kcnk2* mRNA was found in the cartilaginous shell undergoing multisite vacuolization to become the future scala vestibuli (50), whereas, on P0, it was located in the remaining cells bordering the scala vestibuli and forming a multilayer structure covering the RM (*SI Appendix*, Fig. S7*A*). By scanning electron microscopy on P1, P8, and P20, we also observed cells bordering the scala vestibuli and covering RM on P1, which subsequently decreased in number and retracted a few micrometers away from RM (Fig. 5*E*). These cells expressed several causal genes for various deafness forms, including *Eps8l2*, *Gjb2*, *Gjb6*, *Homer2*, *Coch*, *Clic5*, *Dcdc2a*, *Pou3f4*, *Col4a6*, and *Six1*. However, GO term analysis on the genes with the highest degree of differential expression did not identify an enrichment of a particular function (*SI Appendix*, Fig. S7*B* and Dataset S2). To the best of our knowledge, these cells that we name—scala vestibuli border (SVB) cells—have never been identified before as a particular cell type in any histological studies.

### Transcriptomic Insight into the Tonotopic Maturation of the Cochlea.

**Fig. 6.**
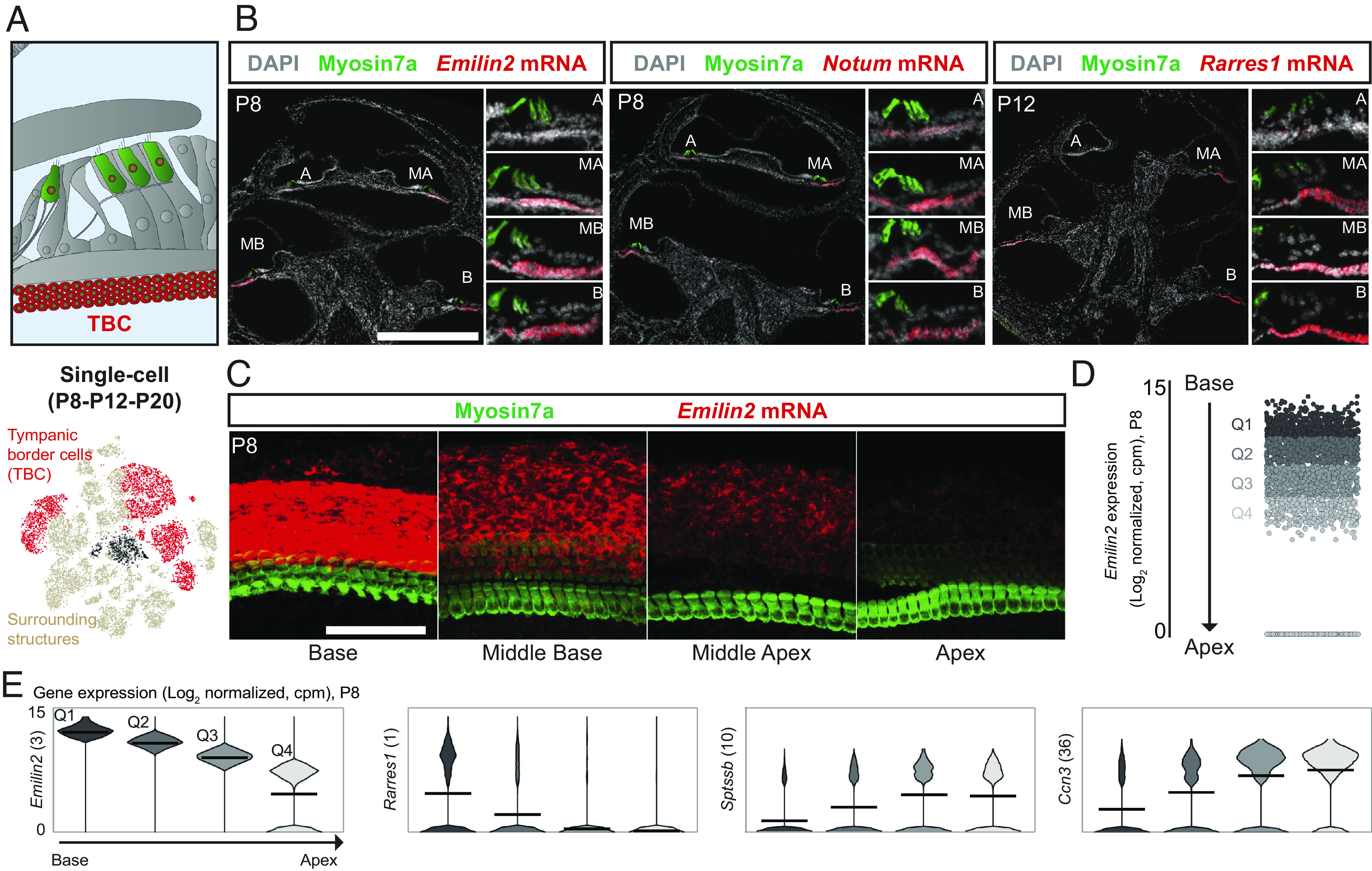
Tonotopic developmental dynamics of tympanic border cells. (*A*, *Top*) Diagram of the cochlea highlighting the TBCs. (*A*, *Bottom*) t-SNE plots of the scRNAseq (P8-P12-P20) datasets for the surrounding structures and TBCs. The black cells correspond to nonassigned cells. (*B*) Z-projection of P8 whole-cochlea cryosections stained with DAPI; immunostained for myosin7a; and stained for *Emilin2, Notum,* and *Rarres1* mRNA with RNAscope (scale bar, 500 µm). (*C*) Z-projection of a P8 whole-mount cochlea, immunostained for myosin7a and stained for *Emilin2* mRNA with RNAscope (scale bar, 100 µm). (*D*) Scatter plots of *Emilin2* expression on P8, split into four quartiles (Q1 to Q4) assumed to represent four subregions of the cochlea. (*E*) Violin plots showing the levels of expression of genes differentially expressed in TBCs (ranked among the most differentially expressed genes, indicated in brackets) classified according to the analysis in *D*. Means are indicated by bars. A: apex; MA: middle apex; MB: middle base; B: base.

The third nonosseus cluster from the surrounding structures displayed strong and specific expression of *Emilin2* (Elastin MicrofibriL INterfacer 2), encoding a major extracellular matrix glycoprotein of the basilar membrane (51). This membrane is a highly specialized extracellular matrix sandwiched between the organ of Corti and a paucicellular connective tissue produced by the underlying TBCs. The basilar membrane, the width, thickness, and stiffness of which vary progressively along the length of the cochlea, acts as a frequency analyzer, generating a tonotopic map with high-frequency sounds detected at the base and low-frequency sounds at the apex of the cochlea (52). The stiffness gradient of the basilar membrane has been proposed to be dependent on Emilin-2, which contributes to extracellular functions and tissue elasticity and is synthesized by the underlying TBCs. Indeed, *Emilin2* inactivation in mice disorders the collagenous fibers running radially within the basilar membrane as well as the supporting matrix and results in abnormal auditory frequency tuning curves (51). By analyzing *Emilin2* mRNA localization using RNAscope assays, we revealed on cochlear P8 cryosections a tonotopic gradient of *Emilin2* expression, with the intensity of expression decreasing from the base to the apex of the cochlea. A similar observation was made for *Rarres1*, identified as a possible tumor suppressor gene (53), and *Notum,* which down-regulates Wnt signaling (54). These three genes were among the most differentially expressed genes in TBCs according to our scRNAseq and snRNAseq data (Fig. 6 *A* and *B* and Dataset S1). The tonotopic gradient of *Emilin2* expression was further confirmed in RNAscope analyses of whole-mount cochlear preparations (Fig. 6*C*).

For the identification of genes with a tonotopic gradient of expression, TBCs collected on P8 were split into four equal-sized groups of cells (quartiles) according to *Emilin2* expression levels and assigned to the base [first quartile (strongest expression level)], middle-base (second quartile), middle-apex (third quartile), and apex [fourth quartile (weakest expression level)] of the cochlea (Fig. 6*D*). TBCs proliferate during the first postnatal week and are arranged in three to four layers of equal thickness along the tonotopic axis on P8. However, shortly thereafter, they undergo changes, as shown by observations from P12 onward, with the basal region containing only one layer, as previously reported (55), precluding direct quantitative interpretations of RNAscope assays at these ages (see P12-20 data in Fig. 6*B* and *SI Appendix*, Fig. S7*A*). The ordering of TBCs on the basis of *Emilin2* expression on P8 was supported by the concomitant strong tonotopic gradient of *Col4a3* and *Col4a4,* encoding type IV collagens, components of the radial collagenous fibers of the basilar membrane. Several genes, including *Rarres1*, *Knck3*, *Notumos*, *Notum*, *Cav1*, *Lypd1,* and *Aox3,* had similar expression profiles to *Emilin2,* with expression gradually decreasing from base to apex. By contrast, the expression gradients of *Sptssb*, *Tnfaip6*, *Ccn3,* and *Tex15* were the opposite of that for *Emilin2* (Fig. 6*E*, Dataset S3, and *SI Appendix*, Fig. S8) (Kruskal–Wallis test followed by NPMC Dunn–Holland–Wolfe test). The molecular pathways involved in the tonotopic features mediated by TBCs were further analyzed by applying GO and molecular network analysis tools (*Materials and Methods*) to genes with a fold-change in expression of more than 1.3 between the base and apex of the cochlea or vice versa. Many of the GO categories identified were related to development, morphogenesis, cell differentiation, and cell death, consistent with the changes in basilar membrane morphology occurring around P8. Several molecular pathways known to be involved in the developmental organization of the cochlea (i.e., at earlier stages) displayed tonotopic expression in TBCs. These pathways comprised the Wnt signaling pathway (*Fzd10*, *Wif1*, *Pip5k1b*), which is involved in early cochlear patterning, including cochlear duct growth (56) and TBC development (55), the ephrin signaling pathway (*Epha5*) involved in tonotopic innervation of the cochlea (57), and the retinoic acid signaling pathway (*Rarres1*, *stra6*) involved in the tonotopic patterning of HCs in chicken (58). Several genes encoding developmental factors, including fibroblast growth factors (*Fgfr2*, *Fgfr4*) and bone morphogenetic growth factors (*Bmp4*, *Smad6*), were also identified (59). Molecular pathways not previously shown to be related to cochlear tonotopy development were also identified. They included the glial cell line–derived neurotrophic factor/*Ret* receptor tyrosinase kinase signaling axis (*Bmp4*, *Robo2*) and the inhibitor of the DNA-binding 2 protein (60). Furthermore, transcription factors differentially expressed in TBCs also displayed a tonotopic gradient similar to that of *Emilin2* (*Gata6, Cux2, Nr1h3, Atoh8,* and *Atf3*) or running in the opposite direction (*Sp5, Foxf2, Dach1, Pbx3, Tbx1, Creb5, Osr1*, *Zic2,* and *Zic5*) (Dataset S3). In conclusion, by combining transcriptomic and RNAscope data, we compiled a list of genes differentially expressed in TBCs with tonotopic gradients of mRNA levels.

### An Atlas of Deafness Gene Expression in the Cochlea.

We then investigated the changes in expression of all the detected genes between P8 and P20 in each cochlear cell type, to assess their individual and relative maturation processes. Remarkably, plots of statistical significance against the fold-change in gene expression (volcano plots) revealed that some of these cell types underwent major changes, suggesting differentiation continuing after P8, whereas others did not (*SI Appendix*, Figs. S9-1 and S9-2 and Dataset S4). Between P8 and P20, the cells from the three stria vascularis layers and most cell types in the surrounding structures, including the SVB cells, RM cells, and osteoblasts, displayed slight-to-moderate changes in gene expression. By contrast, unlike HCs, cell types such as SCs, TBCs, fibrocytes, osteocytes, SS1, and SS2 cells, displayed major changes (*SI Appendix*, Figs. S9-1 and S9-2). Fibrocytes displayed the most marked changes, with a strong downregulation of key transcription factors, such as Pou3f4 and MafB, and of several structural proteins (Col11a1, Col11a2, Tectb), but an increase in the expression of otospiralin, and the gap junction proteins Gjb2 and Gjb6. Some of the changes revealed here can be tentatively correlated with known changes in cell structure, morphology, and function, such as the formation of some OHC hair bundle links. However, tectorial membrane maturation, which involves proteins expressed by SCs (Tecta, Tectb, Ceacam16, Otoa, Otog, and Otogl) (61), and other changes highlight an acquisition of functions yet to be elucidated.

**Fig. 7.**
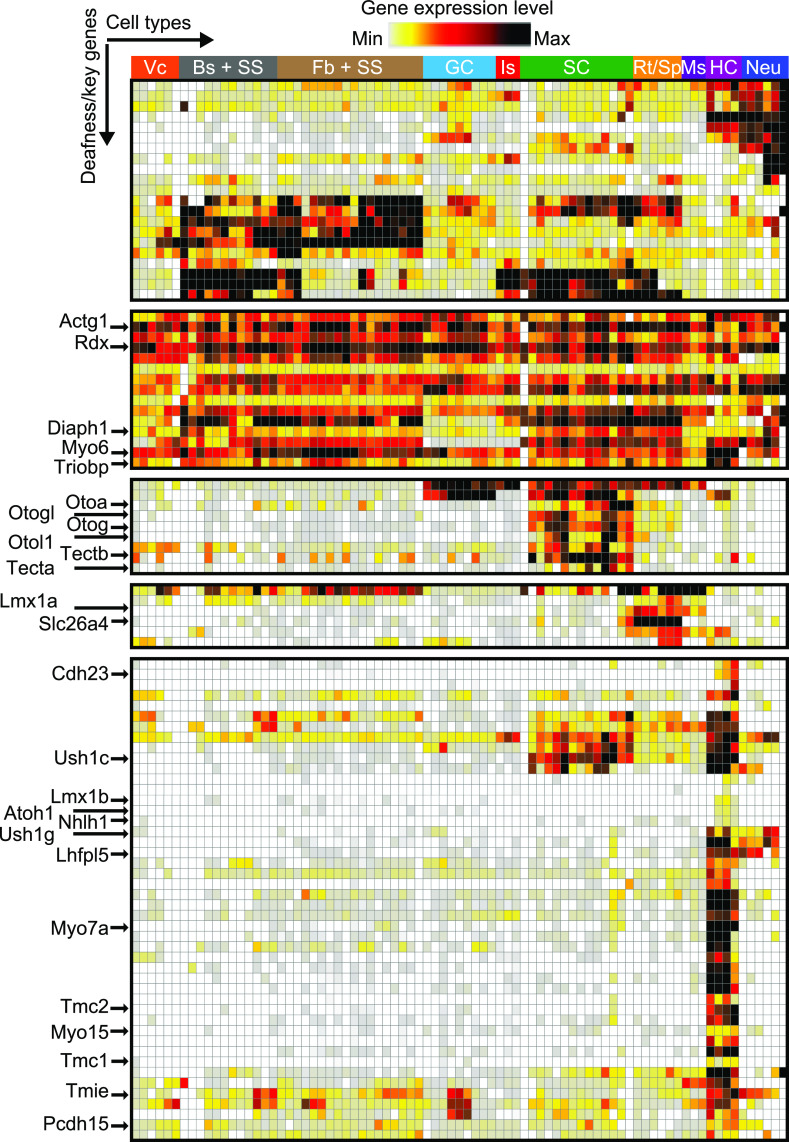
Cochlear cell expression pattern of nonsyndromic deafness genes. Hierarchical clustering of cochlear cell types and 195 detected deafness/key regulatory genes in scRNAseq (P8, P12, P20) and snRNAseq (P8, OHCs only) data. Highlighted genes are indicated by an arrow. In total, 97 of the 195 deafness/key genes are shown for display purposes. The black frames indicate when genes were removed. Vc: vascular cells, Bs + SS: basal stria cells + surrounding structures, Fb + SS: fibrocytes + surrounding structures, GC: glial cells, Is: intermediate stria cells, SC: supporting cells, Rt/Sp: root cells and spindle cells, Ms: marginal stria cells, HC: hair cells, Neu: neurons.

Next, we investigated the possible clustering of genes key to cochlear development and function according to their temporal and spatial patterns of expression, to decipher the molecular basis of the regulation of their expression and associated gene regulatory networks. We therefore analyzed the cochlear cell expression patterns of 120 detected genes involved in isolated forms of deafness and 75 detected key genes for cochlear development and function (62) on P8, P12, and P20. We first applied a similarity matrix based on the hierarchical clustering of cochlear cell types according to the expression of these genes based on scRNAseq (P8, P12, and P20) and snRNAseq (P8, OHCs only) data (*SI Appendix*, Fig. S10-1). Cells of the same cell type but different ages clustered together, except for osteocytes (for which P8 cells clustered separately from P12 and P20 cells) and the various types of SCs, which were intermingled. We then performed hierarchical clustering analysis on these genes (see the *y* axis in Fig. 7 and see *SI Appendix*, Figs. S10-2 and S10-3 for exhaustive clustering) and found that they formed several major clusters. For example, many genes involved directly or indirectly in HC mechanoelectrical transduction, including those encoding the channel subunits Tmc1 and Tmc2, the transmembrane proteins Tmie and Lhfpl5, the molecular motors Myo7a and Myo15a, and the submembrane protein Ush1g, clustered together in a single group of genes expressed only in HCs. However, *Ush1c,* which also encodes a mechanoelectrical transduction component, harmonin, formed another cluster with other genes expressed in HCs, all of which were also expressed in SCs. Similar clustering was observed for *Pcdh15,* encoding the lower part of the tip-link, which is also expressed in glial precursor cells. Genes involved in a particular cochlear function involving several cell types also clustered together. Examples include tectorin-α and tectorin-β (*Tecta*, *Tectb*), noncollagenous tectorial membrane proteins (*Otol1*) (63), and proteins involved in the attachment of the OHC hair bundle to this membrane, such as otogelin (*Otog*) and otogelin-like (*Otogl*), all of which are expressed in several types of SCs. Notably, several genes displayed unexpected expression profiles as *Myo6* which had a pleiotropic expression; *Gjb2* that was expressed in the SVB cells and the SS2 cells of the modiolus; and *Otol1* (Otolin-1) that clustered with *Otog*, *Otogl,* and *Tectb* due to its expression in all SC types. The 195 genes analyzed formed about 20 clusters of genes, for which the role of transcription factors expressed by the corresponding cells can now be explored.

## Discussion

The transcriptomic atlas presented here provides a deep profiling of cochlear cell types, highlighting the genes with the strongest differential expression at various time points in postnatal differentiation. It also provides additional transcriptomic characterization for cochlear cell types for which transcriptomic data to date were not distinctive including various subcategories of SCs and glial cells. It identifies additional specific markers improving the identification of poorly characterized cochlear cell types, such as the cells of RM and the basal stria layer. Finally, it reveals previously undescribed cochlear cell types. Two of these cell types localized to the modiolus, a spongy bone structure, and had bone cell–like transcriptomic profiles that nevertheless differed from those of preosteoblasts, osteoblasts, and osteocytes. The third cell type—SVB cells—is characterized by a unique transcriptomic signature different from that of other cochlear cells, including fibrocytes present in the same area of the cochlea. Based on scanning electron microscopy and RNAscope studies, we propose that SVB cells may maintain the structure of the thin RM during the vacuolization of cartilaginous shell cells, resulting in a tunneling of the scala vestibuli, with a small cluster of cells persisting at the base of RM at later stages.

The molecular mechanisms underlying the establishment of the tonotopic gradient in the biophysical characteristics of the basilar membrane remain unknown. This is an issue of the utmost physiological relevance, as it underlies passive frequency analysis by the cochlea (52). This atlas provides insight into the postnatal establishment of the tonotopic features of the basilar membrane, morphologically characterized by radial collagenous fibers, which increase in thickness and density, from the base to the apex, during maturation (64). Based on the tonotopic gradient of *Emilin2* expression, which was paralleled by the gradients of type IV collagen expression, as shown here at single-cell level by transcriptomic analysis in combination with RNAscope assays, we were able to establish a list of genes with mRNA levels following a tonotopic gradient at single-cell level, either decreasing from the base to the apex of the cochlea as for *Emilin2,* or decreasing from the apex to the base of the cochlea. Some of the genes differentially and tonotopically expressed in the TBCs encode proteins of the signaling pathways involved in the early patterning of the otocyst, such as the Wnt pathway for patterning of the dorsoventral axis (65), suggesting that they are also essential for tonotopic patterning of the basilar membrane. Remarkably, some of the 14 transcription factors following a tonotopic gradient of expression in TBCs had already been identified as encoded by genes responsible for deafness in humans and/or mice. These genes include *Tbx1,* which is involved in inner ear morphogenesis (66), *Dach1* in stria vascularis development (67), *Foxf2* and *Zic2* in cochlear formation and elongation (68, 69), and *Atoh8* encoding a bHLH transcription factor. Our data showing that *Atoh8* is differentially expressed in the TBCs during the postnatal period may provide clues to the basis of the deafness of unknown origin in *Atoh8* mutant mice (70). Each of these transcription factors provides a molecular entry point for exploring the gene regulatory networks involved in establishing the tonotopic properties of the basilar membrane.

This transcriptomic atlas will benefit auditory research by accelerating the identification of candidate target cell types in the various genetic forms of deafness. It will also provide a highly valuable source of information for the design of gene therapy agents and vectors for preventing or curing deafness. Many genetic forms of deafness have been studied by focusing on a particular cochlear cell type or group of cell types thought to be sufficient to account for the auditory phenotype. Our atlas, by pinpointing additional cochlear cell types expressing some deafness genes, draws attention to their possible involvement in auditory phenotypes, providing a potential explanation for the partial or short-lived rescue observed in some preclinical gene therapy trials performed on P0-P2 (71). This atlas provides a glimpse of the efforts that will be needed to design vectors specifically targeting certain combinations of cell types at appropriate time points.

Previous major single-cell transcriptomic studies have focused on the embryonic and postnatal prehearing periods up until P7 (15), particular cell types (17) or were based on a few thousand cells (72). All but one of the proofs of concept for gene therapy obtained in mice to date, the exception being that targeting otoferlin (4), were obtained exclusively for postnatal interventions performed before P7. They therefore demonstrate the feasibility of preventing hearing impairment in the immature cochlea. However, it remains particularly challenging to cure hearing impairment through interventions on the mature mouse cochlea (73, 74), an essential step on the road to developing early postnatal gene therapy interventions for the already-mature human cochlea. By showing that some deafness genes have low levels of expression in some cochlear cell types, increasing only in a given cochlear cell type at a particular stage, this transcriptomic atlas should help to improve the design of gene therapy agents and vectors. Furthermore, our data suggest that the genes underlying genetic forms of deafness may cluster together on the basis of the similarity of their expression patterns. The definition of about 20 clusters of genes by this procedure should accelerate the adaptation of gene therapy agents for a particular causal deafness gene to other genes from the same group, by at least initially bypassing the need to develop promoters and viral capsids.

In conclusion, the transcriptomic signatures of cochlear cell types defined in our atlas will serve as a basis for deciphering the role of causal or predisposing deafness genes and the associated normal and pathogenic pathways. This atlas will be particularly useful for identifying overlooked candidate cochlear cells affected by particular deficits in some monogenic forms of deafness. It will be also instrumental to deciphering the signaling pathways and gene regulatory sequences and networks present in cochlear cells, for the development of effective and safe gene therapy procedures.

## Materials and Methods

Animal experiments were performed in accordance with French and European regulations for the care and protection of laboratory animals (EC Directive 2010/63, French Law 2013–118, February 6, 2013), under authorizations from the Institut Pasteur’s ethics committee for animal experimentation. For details of single-cell/nucleus isolation, sequencing, and analysis, as well as RNA in situ hybridization assays, immunohistofluorescence, and microscopy, see *SI Appendix, Materials and Methods*.

## Supplementary Material

Appendix 01 (PDF)

Dataset S01 (XLSX)

Dataset S02 (XLSX)

Dataset S03 (XLSX)

Dataset S04 (XLSX)

Dataset S05 (XLSX)

## Data Availability

All study data related to this work are available in the main text and supporting information as well as the gEAR Portal (https://umgear.org/) in the datasets “scRNA-seq—P8, P12, P20 mouse cochleae (Michalski, 2023)” and “snRNA-seq—P8 mouse cochleae (Michalski, 2023).”
