# Supplementary material for: Single-cell transcriptomic profiling of the mouse cochlea: An atlas for targeted therapies
Source: Proc Natl Acad Sci U S A. 2023 Jun 20;120(26):e2221744120. doi: 10.1073/pnas.2221744120 (PMC10293812; doi:10.1073/pnas.2221744120)
Supplement: Supplementary file 1 — Appendix 01 (PDF) [file pnas.2221744120.sapp.pdf]

PNAS

Supporting Information for

Single-cell transcriptomic profiling of the mouse cochlea: an atlas for targeted therapies

Philippe Jean[a], Fabienne Wong Jun Tai[b], Amrit Singh-Estivalet[b], Andrea Lelli[b], Cyril Scandola[c], Sébastien Megharba[d], Sandrine Schmutz[d], Solène Roux[b], Sabrina Mechaussier[a], Muriel Sudres[b], Enguerran Mouly[b], Anne-Valérie Heritier[b], Crystel Bonnet[b], Adeline Mallet[c], Sophie Novault[d], Valentina Libri[d], Christine Petit[b,e,1,2], and Nicolas Michalski[a,1,2]

[a]Institut Pasteur, Université Paris Cité, INSERM, Institut de l'Audition, Plasticity of Central Auditory Circuits, F-75012 Paris, France
[b]Institut Pasteur, Université Paris Cité, INSERM, Institut de l'Audition, Auditory Therapies Innovation Laboratory, F-75012 Paris, France
[c]Institut Pasteur, Université Paris Cité, Ultrastructural BioImaging, F-75015 Paris, France
[d]Institut Pasteur, Université Paris Cité, Cytometry and Biomarkers, F-75015 Paris, France
[e]Collège de France, F-75005 Paris, France

[1]To whom correspondence may be addressed. Email: christine.petit@pasteur.fr or nicolas.michalski@pasteur.fr

[2]C.P. and N.M. contributed equally to this work.

This PDF file includes:

Materials and methods

Figures and figure legends S1 to S10

Legends for Datasets S1 to S5

SI References

MATERIALS AND METHODS

Animals

C57BL6/J wild-type mice were purchased from Janvier Laboratories.

Single cell/nucleus isolation and sequencing

The cochleae of P8, P12, and P20 animals were extracted by removing the osseous labyrinth and dissected in phosphate-buffered saline (PBS) solution. Briefly, after removal of the osseous shell, whole cochleae were microdissected and then subjected to enzymatic dissociation (Adult Brain Dissociation kit, 130-107-677, Miltenyi Biotec) (Fig. S1A) and mechanical trituration with a gentleMACS dissociator (Miltenyi Biotec). The resulting suspension was filtered sequentially through 70 µm and 40 µm meshes and then sorted by flow cytometry on the basis of size (forward scatter), granularity (size scatter), and viability (Fig. S1B) with a BD FACSAria III cell sorter (BD Biosciences) in PBS with 0.04% bovine serum albumin (BSA). To overcome the biases of cell sorting, which are dependent on the viability and morphology of a given cell type, we complemented this scRNAseq approach by generating a transcriptomic dataset for single nuclei, which are morphologically similar between the different cell types and the preservation of which is less dependent on tissue health. The snRNAseq dataset was obtained on P8 (Fig. S1B) but not at older ages, as the number of harvested viable nuclei was not high enough for subsequent sequencing and analysis. For snRNAseq, we used the “Nucleus isolation from cell suspensions for ScRNAseq protocols” (CG000124 RevF, 10x genomics). DAPI-positive nuclei (1/10,000, incubation for 10 minutes) were sorted on the basis of forward scatter and size scatter. The sorted single-cell/single-nucleus suspension was treated (approximatively 3 h after animal sacrifice) according to the “Chromium Next GEM Single Cell 3’ v3.1 protocol” (CG000204 Rev D, 10x genomics) in accordance with the manufacturer’s instructions, for library construction. The quality of the libraries was checked with an Agilent 2100 Bioanalyzer and the High-Sensitivity DNA kit (Agilent), and the libraries were then sequenced with a HiSeqX Illumina sequencer at a depth of 50 000 reads per cell (Macrogen).

Single cell/nucleus RNA sequencing analysis

Sequencing data were processed and analyzed with Partek Flow analysis software (Partek, St. Louis, Missouri). The STAR 2.7.8a algorithm was used to align sequences with the whole mouse genome index and the mm10 assembly. The duplicated Unique Molecular Identifiers were removed and barcodes were quantified with the Ensembl100 annotation model. Intronic reads were included for snRNAseq.

For scRNAseq, the following criteria for the selection of healthy cells and exclusion of duplicates were applied: 500-6000 genes, 1250-25000 counts, 0-8% mitochondrial reads and 0-10% ribosomal reads. Gene expression was normalized in counts per million (by dividing by the total number of mapped reads per sample and multiplying by 1×10^6). A count of 1 was then added before log_2 transformation of the data. Genes not expressed in 99.9% of cells were filtered out, resulting in 22,588 gene entries being considered in subsequent analyses. Principal component analysis focusing on the most variable features was performed and the top 15 principal components selected before data visualization with a t-distributed stochastic neighbor-embedding (t-SNE) representation for dimension reduction, with a Euclidean distance metric with 30 as perplexity and 1000 iterations. Cells were classified into cell types manually, based on their clustering and the combined expression patterns of several markers already known and published or characterized by our RNAscope assays. The classification was strengthened further by comparing the findings for the top 200 most differentially expressed genes between these cell types with published scRNAseq data for the cochlea. The cells without genes displaying high levels of differential expression (i.e. those without a clear transcriptomic signature) could not be classified and were therefore labeled as "nonassigned". The high heterogeneity in cell size, morphology and number introduced a notable bias into the proportions of the various cell types sorted by flow cytometry (Fig. S1B) and therefore the relative proportions of the cell populations on the t-SNE plots in scRNAseq do not reflect their respective contributions observed on histological preparations (Fig. 1B). Since all the characterized cell types were detected in each sample, excluding any obvious batch effect due to the various biological replicates analyzed (Fig. S2), no batch effect corrections were applied.

For snRNAseq, we applied the following criteria: 400-3000 genes, 500-5000 counts, 0-4% mitochondrial reads and 0-10% ribosomal reads. The same PCA and t-SNE algorithm parameters were used to visualize the cells as for scRNAseq, and the same cell-type classification method was employed. Erythrocytes were detected in the single-nucleus sequencing dataset. As erythrocytes have no nuclei, this observation suggests that whole cells may have escaped the filtering and were sorted with the nuclei. Indeed, on analysis of the snRNAseq t-SNE plots, we occasionally observed two well-separated clusters, which therefore had different transcriptomes, but were of the same cell type, suggesting that a fraction of whole cells had been collected with the nuclei. Cluster duplication for a given cell type was not due to batch effects (see Fig. S2) and was not observed in the scRNAseq dataset. Moreover, for each cell type, a comparison of the 500 most differentially expressed genes between the two corresponding snRNAseq and scRNAseq clusters on P8 showed that the smaller snRNAseq cluster had the strongest transcriptomic correspondence to the scRNAseq data cluster. We

therefore considered this smaller cluster to be the one containing the unwanted whole cells in our snRNAseq data and we removed this cluster from the analysis.

The total number of genes detected for the whole atlas was similar for the two approaches: 22 588 genes for scRNAseq and 20 344 genes for snRNAseq. However, an average expression of 2068 genes per cell was detected across all ages with scRNAseq in comparison to 828 genes for snRNAseq.

RNA *in situ* hybridization and immunohistofluorescence assays

The cochleae were extracted in cold freshly prepared PBS solution and fixed by incubation in 4% paraformaldehyde (PFA) for 1 h at room temperature (RT). For cryosections, the organs were decalcified by incubation in 0.35 M EDTA, pH 7.5 at 4°C for 24 h for the P8 samples, 48 h for the P12 samples and 72 h for the P20 samples. The cochleae were then post-fixed by incubation in 4% PFA for 1 h, and were then incubated overnight in 20% sucrose at 4°C. They were embedded in optimal cutting temperature (OCT) compound (VWR International), and the resulting blocks were frozen in Tissue Tek Cryomold Intermediate (Q93740, Interchim) in liquid nitrogen and stored at -80°C. The cochlear blocks were cut into 10-12 µm-thick slices on a cryostat (CryoStar NX70, Epredia), and allowed to dry for 1 h at RT before being stored at -20°C. For RNA *in situ* hybridization assays, the RNAscope kit (RNAscope Multiplex Fluorescent Reagent Kit v2 Assay, catalog no. 323100) purchased from Advanced Cell Diagnostics (Bio-Techne SAS, Rennes) was used according to the manufacturer's instructions. The OCT was removed, and slices were heated for 30 minutes at 40°C and post-fixed by incubation in 4% PFA for 15 minutes at RT. For whole-mount RNAscope assays, the organs of Corti were microdissected. Both types of preparation were washed in H_2O, dehydrated in a successive series of ethanol solutions and dried for 30 minutes at 40°C. The slices (but not the whole mounts) were incubated with hydrogen peroxide and heated in a HybEZ oven for 5 minutes at 99°C. The tissues were incubated with Protease Plus solution in the hybridization oven at 40°C for 30 minutes and were then hybridized with the target probes at 40°C for 2 h. Each target probe contained a mixture of short oligonucleotides designed to bind to a specific target mRNA and detectable in one of three fluorescence channels (see Dataset S5 for full list of probes), C1, C2 or C3. Different fluorophores were assigned to the C1, C2 and C3 channels, depending on the Opal™ dye (Akoya Biosciences) selected for the channel concerned. The tissue was washed several times and then subjected to sequential hybridization procedures with the RNAscope Multiplex Fl V2 Amp1, Amp2 and Amp3 and horseradish peroxidase, to amplify the signal. The fluorescent probes Opal dye 488, 570 or 649 (1/1000 dilution) were then added for channel C1, C2 or C3 labeling. If additional immunofluorescence staining was required, the tissues were then incubated in a blocking solution (0.3% Triton-W100, 1% BSA, 20% goat serum) for 1 h at RT, and then with the primary antibody overnight at 4°C, followed by the secondary antibody for 2 h at RT. DAPI

(SIGMA, MBD 0015-1 mL) was then added at a dilution of 1/1000, for 10 minutes at RT. The tissues were mounted on coverslips with ProLong Gold Antifade Mountant (Invitrogen, Ref: P10144). The antibodies used were: a rabbit primary antibody directed against Myo7a (1/200 Proteus Ref:25-6790, USA), and a goat anti-rabbit Alexa fluor 488 (1/500, Ref: A32731 Invitrogen)-conjugated secondary antibody.

Serial electron microscopy

For electron microscopy, samples were fixed by incubation in 2% PFA and 2% glutaraldehyde in PBS for 2 h at RT. P20 cochleae were washed in PBS and decalcified by incubation in 0.5 M EDTA-PBS for 48 h. All samples were then washed in water, post-fixed by incubation in osmium tetroxide 2% for 1 h, washed in water, and dehydrated in a series of acetone solutions at concentrations from 70% to 100%. Samples were embedded in Spurr resin (EMS), which was allowed to polymerize for 48 h at 60° C. Ultrathin sections (100 nm) were cut with a Leica Ultracut S microtome, stained with uranyl acetate and lead citrate, and sputtered with 7 nm carbon. Sections were observed under an IT700HR (JEOL) scanning electron microscope (SEM) operating at 5kV with an-in lens back-scattered electron detector. The contrast of the images was inverted with the SEM software.

Fluorescence microscopy

Images were acquired with an LSM 900 Airyscan (Zeiss, Oberkochen, Germany), with excitation lasers at 390, 470, 550, and 640 nm, with either a 0.8 numerical aperture (NA) 10x air objective, or a 1.4 NA 63x oil immersion objective. Images were Z-projected and adjusted for brightness and contrast with Fiji software.

Statistical and data analysis

The most differentially expressed genes were identified by Student's *t*-tests on the classified cell types with Partek Flow analysis software, comparing each group, one-by-one, with all the other groups together. The upregulated genes were identified as markers, and were sorted on the basis of their *p*-values (Dataset S1). For multiple comparisons (Fig. S8), values of $p < 0.05$ were considered significant in Kruskal–Wallis (K–W) tests followed by non-parametric multiple-comparison tests (NPMC, Dunn-Holland-Wolfe test) for non-normally distributed data and datasets of equal size, in Igor Pro 6.3 (Wavemetrics). All genes were taken into account for the hierarchical clustering presented in Figs. 5 and S3. The average linkage method was used to determine cluster distance metrics and the Euclidean method was used to determine point distance metrics in Partek software. For Fig. 7, hierarchical clustering tools, such as Morpheus (software.broadinstitute.org/morpheus) and Clustergrammer (maayanlab.cloud/clustergrammer) (1), were used. For bubble plots presented in Figs 1, 3 and S6,

mean gene expression level and the percent of cells with a gene expression value above 0 were considered. For Gene Ontology Enrichment Analysis, the 200 most differentially expressed genes in a given cell type were analyzed with the open-source python-based library GOATOOLs (tanghaibao/goatools on github) (2). For the tonotopy study (Fig. 6), the biological processes and molecular pathways were analyzed with online gene ontology (http://geneontology.org/) (3) and molecular network analysis tools (https://cytoscape.org/) (4). The frequency of genes for a particular GO term was compared to the background frequency. The uncorrected *p*-value was then calculated in a Fisher's exact test, with correction for false discovery rate according to the Benjamini/Hochberg procedure.

Figure S1

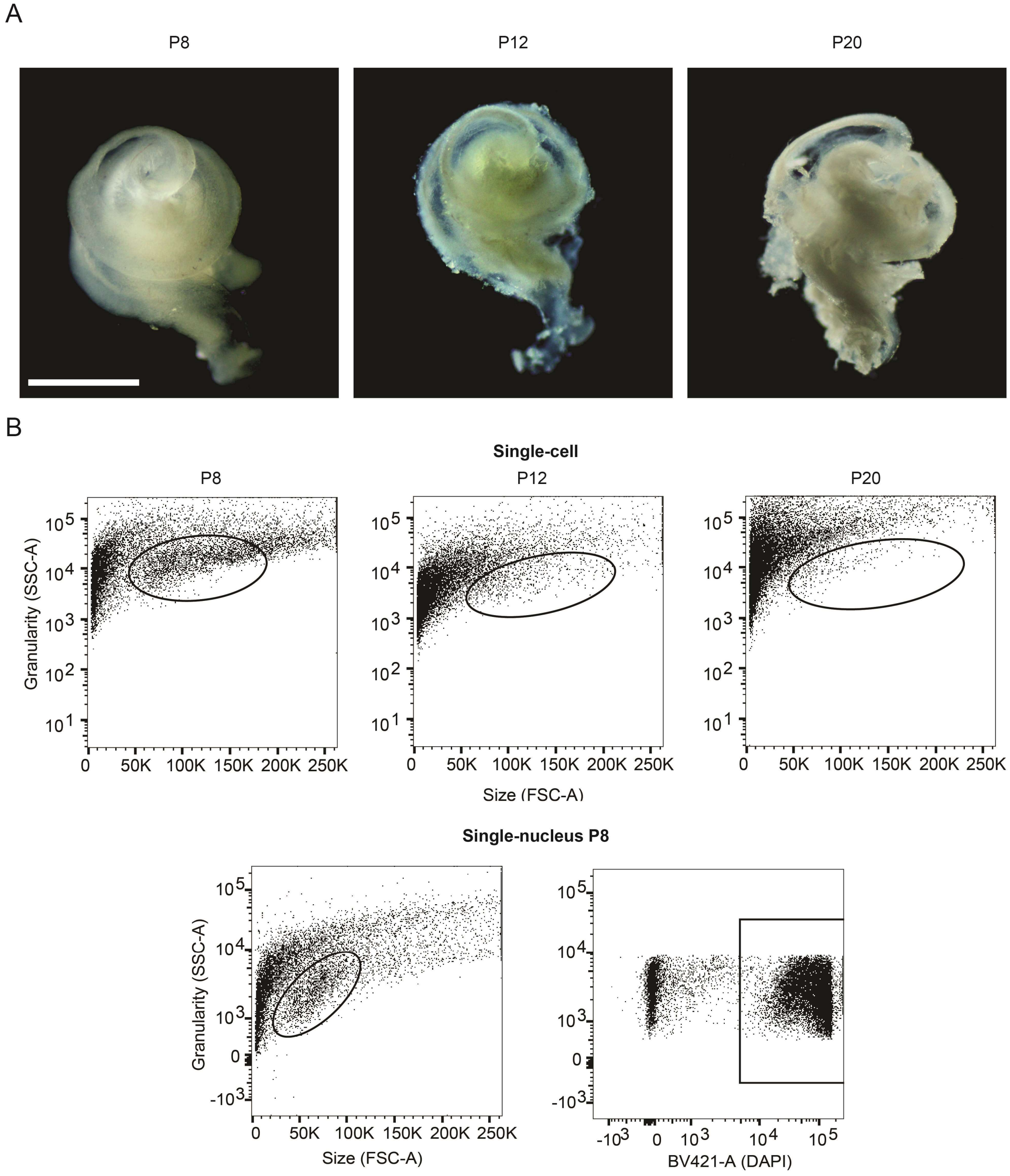

Fig. S1. Dissection of the cochlea and cell/nucleus sorting. (*A*) Whole cochleae at P8 (left), P12 (middle) and P20 (right) after removal of the osseous labyrinth. Scale bar: 500 µm. (*B*) Top row: Cell-sorting profiles on P8, P12 and P20, based on side scatter (SSC-A) as an indicator of particle granularity, and forward scatter (FSC-A) as an indicator of particle size. The gates for selecting the live cells are indicated by the ellipses. Bottom row: Nucleus sorting profile on P8; an additional gate was added to select only nuclei positive for DAPI (BV421-A).

Figure S2

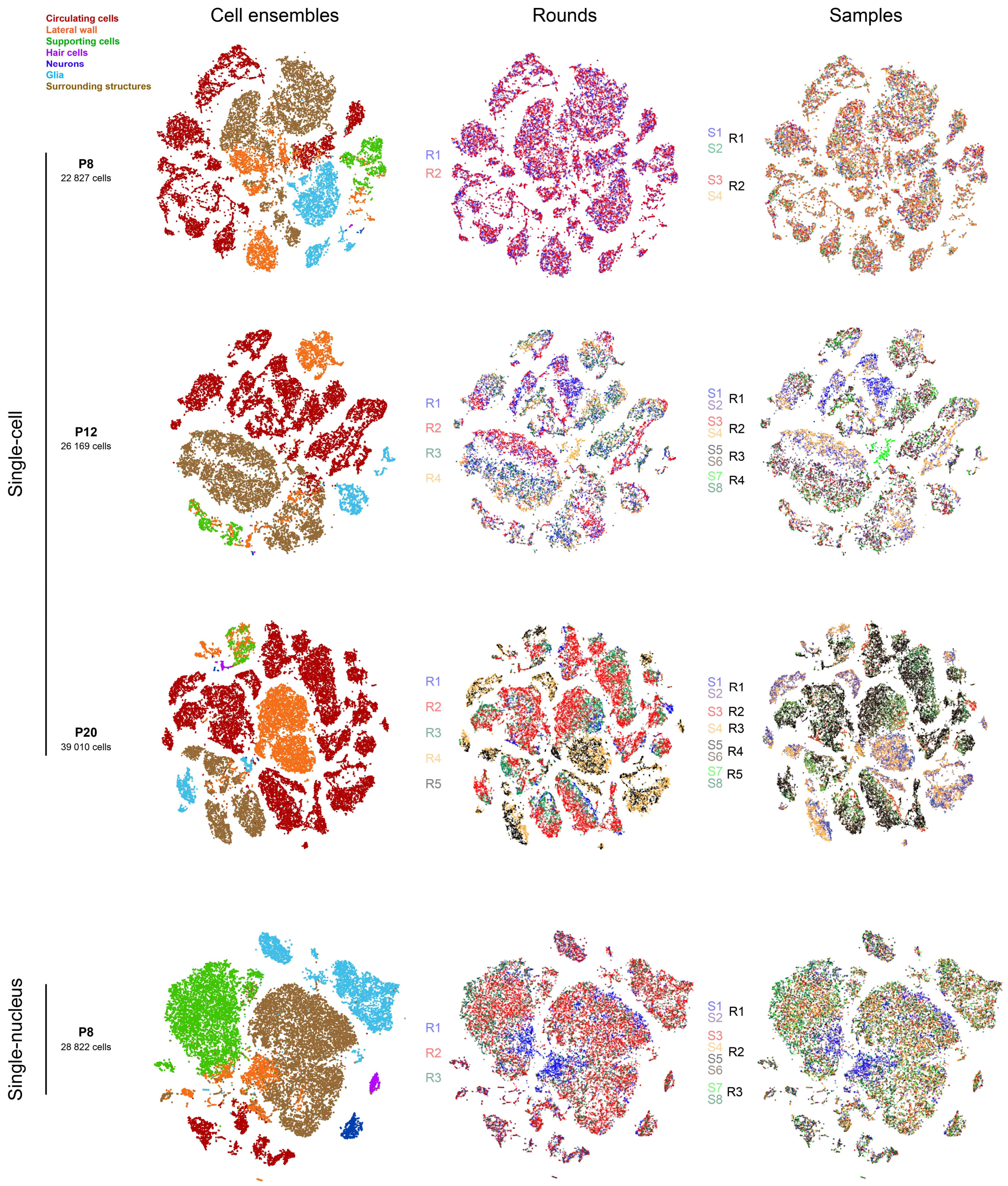

Fig. S2. Batch effect analysis. T-SNE plots color-coded by cell ensemble (first column: circulating cells (red), lateral wall (orange), supporting cells (green), hair cells (purple), neurons (dark blue), glia (light blue), and surrounding structures (brown)), round of experiments (second column: each round of the experiment is associated with a day of experimentation comprising dissection, dissociation, cell sorting and encapsulation/RT-PCR), and sample (third column: each sample consists of 9 animals) according to the isolation technique (cell or nucleus) and age (P8-P12-P20).

Figure S3

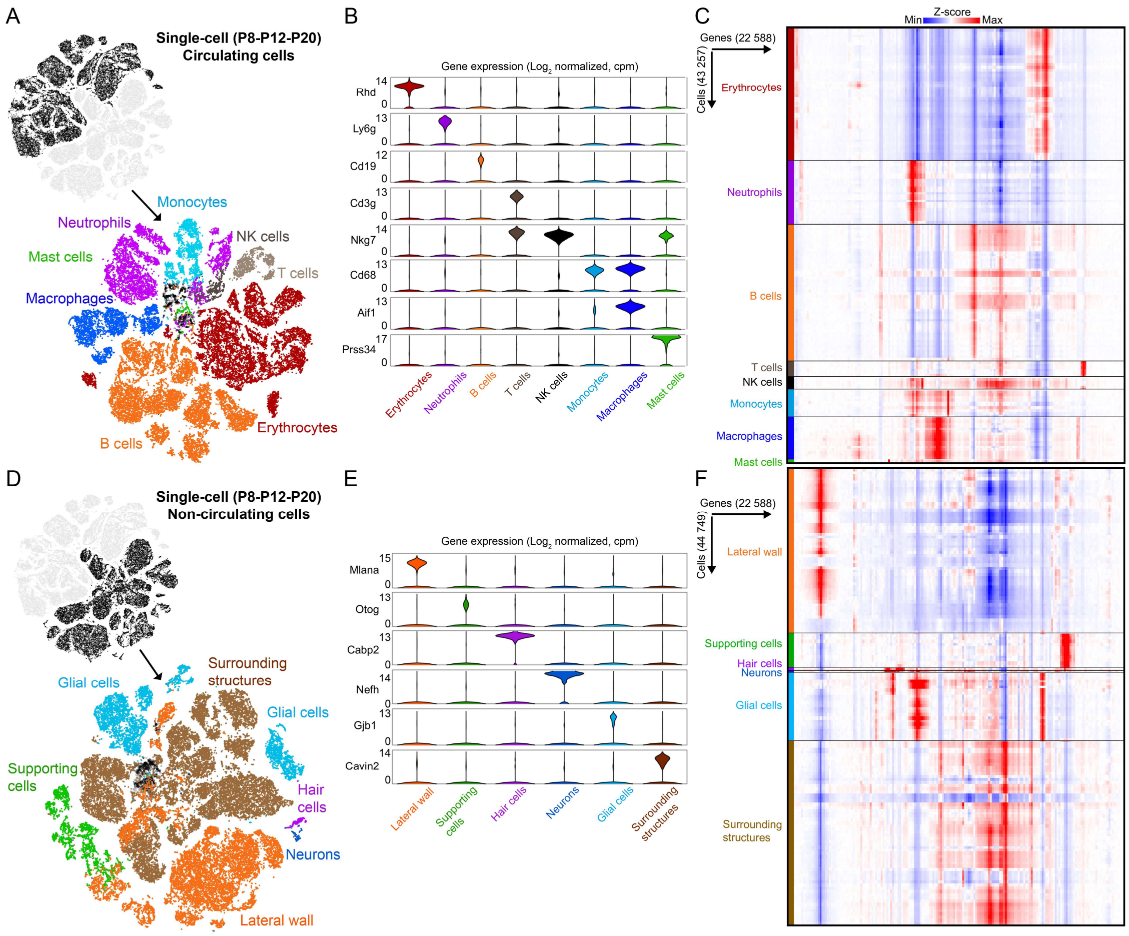

Fig. S3. Capturing the full cell-type diversity of the cochlea. (*A*) Global t-SNE plot of the scRNAseq (P8-P12-P20) datasets, with the circulating cells (in black) reclustered and classified as erythrocytes (red), neutrophils (violet), B cells (orange), T cells (light brown), NK cells (dark brown), monocytes (light blue), macrophages (blue) and mast cells (green, in the middle of the plot). Nonassigned cells are shown in black in the annotated t-SNE. (*B*) Violin plots showing expression levels (counts per million, log_2-normalized) for a subset of the genes used to classify the circulating cell types. (*C*) Hierarchical clustering of the circulating cells with all the detected genes clustered on the *x* axis, and cell types on the *y* axis. Gene expression levels are indicated by the *Z*-score, with high levels of expression in red, intermediate levels in white and low levels of expression in blue. (*D*) Global t-SNE plot of scRNAseq (P8-P12-P20) datasets for the non-circulating cells (in black) reclustered into main ensembles as lateral wall cells (orange), supporting cells (green), hair cells (violet), neurons (dark blue), glial cells (light blue), and surrounding structures (brown). The nonassigned cells are shown in black in the annotated t-SNE. (*E*) Violin plots showing expression levels (counts per million, log_2-normalized) for a subset of genes used to classify the non-circulating cell types. (*F*) Hierarchical clustering of the non-circulating cells as in (*C*).

Figure S4

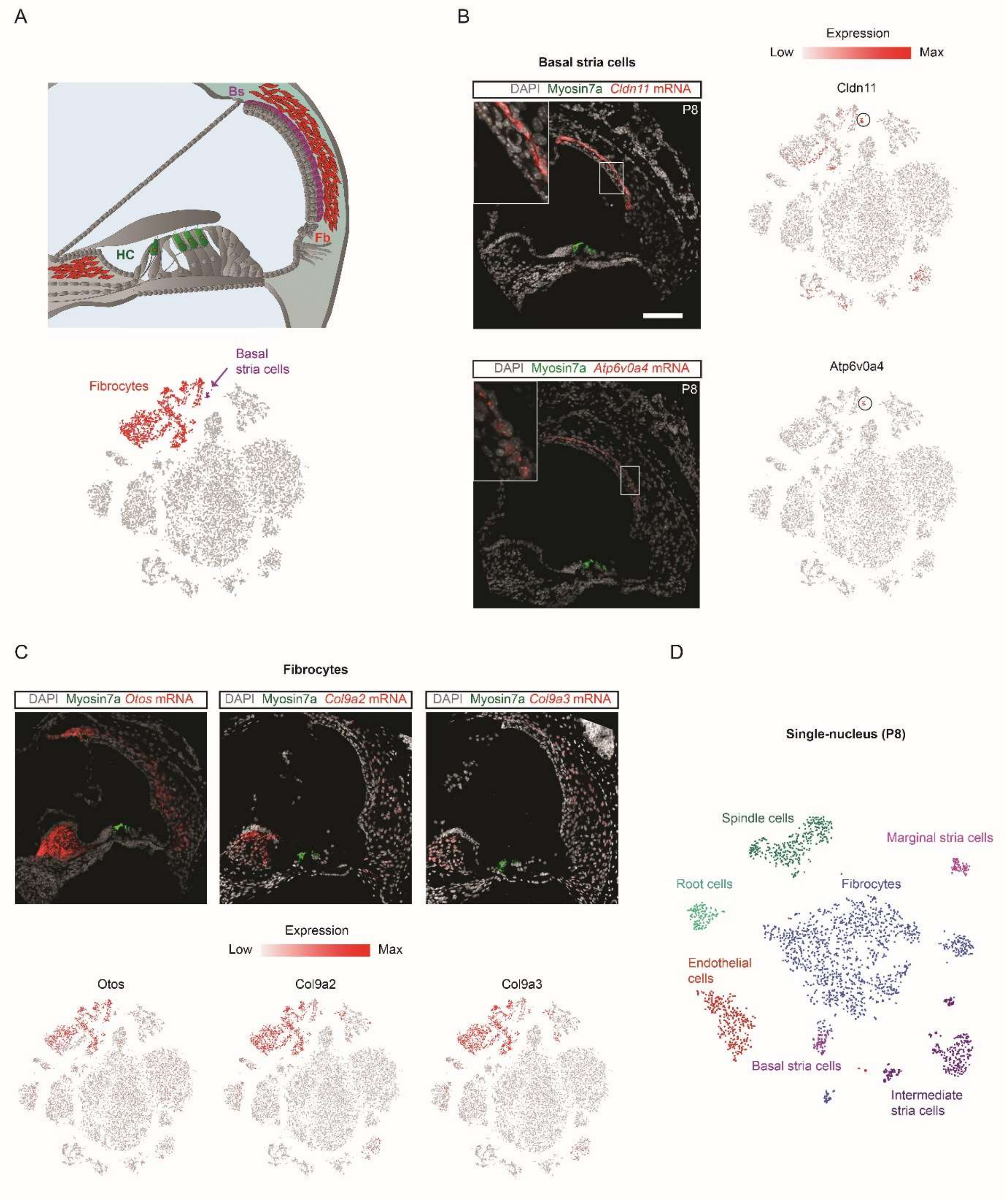

Fig. S4. Transcriptomic characterization of the fibrocytes and basal stria cells. (*A*) Top: Diagram illustrating the cochlea and highlighting the fibrocytes (red), the basal stria cells (violet) and the hair cells (green). Bottom: t-sne plot of scRNAseq data (P8-P12-P20) from the lateral wall cells, according to the same color code. (*B*) Top: Z-projection of a P8 cochlea cryosection focusing on the scala media compartment stained with DAPI (gray), immunostained for myosin7a (green), and stained for *Cldn11* mRNA and *Atp6v0a4* mRNA with RNAscope (red). The inset shows a magnification of the three stria vascularis layers in the white frame. Bottom: T-SNE plots of scRNAseq data (P8-P12-P20) from the lateral wall cells indicating the expression levels of the *Cldn11* and *Atp6v0a4* transcripts. *(C)* Top: Z-projection of a P8 cochlea cryosection focusing on the scala media compartment stained with DAPI (gray), immunostained for myosin7a (green), and stained for *Otos*, *Col9a2* and *Col9a3* mRNA with RNAscope (red). Bottom: T-SNE plots of scRNAseq data (P8-P12-P20) from the lateral wall cells indicating the expression levels of the *Otos*, *Col9a2* and *Col9a3* transcripts. (*D*) t-SNE plot of the snRNAseq (P8) dataset for lateral wall cells.

Figure S5

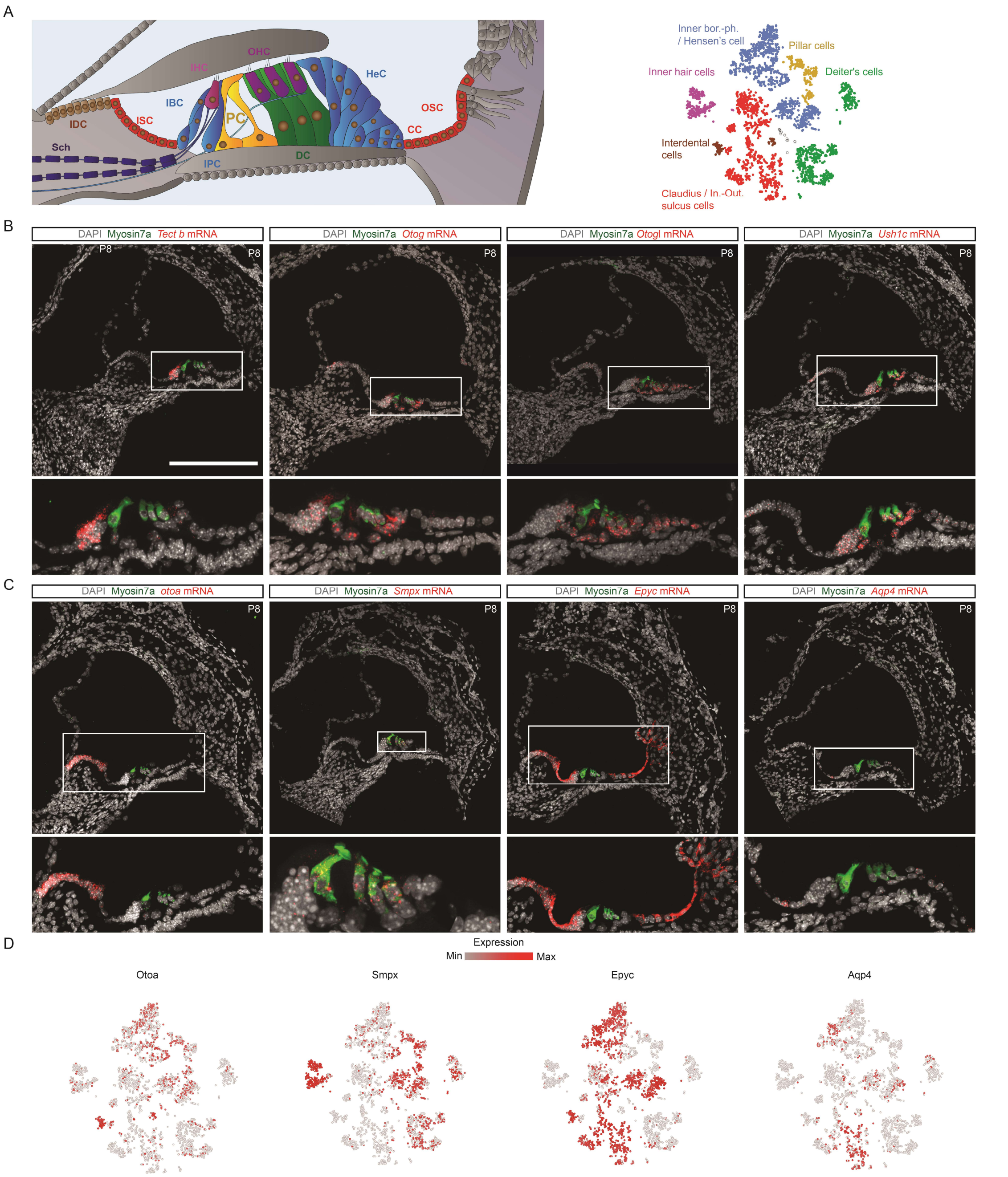

Fig. S5. Transcriptomic characterization of the supporting cells. (*A*) Left: Diagram of the organ of Corti. The transcriptomic data identified one group of cells corresponding to the inner border (IBC), inner phalangeal (IPC), Hensen's (HeC), shown in blue, another group of cells corresponding to inner-outer sulcus (ISC-OSC) and Claudius' cells (CC), shown in red, and individual cell types, such as Deiter's cells (DC) in green, pillar cells (PC) in yellow and interdental cells (IDC) in brown. Right: t-SNE plot of the organ of Corti with the same color code as in diagram. The black circles correspond to nonassigned cells. (B) Z-projection of P8 cochlea cryosections, focusing on the scala media compartment and stained with DAPI (gray), immunostained for myosin7a (green), and stained for *Tectb*, *Otogl*, *Otog* and *Ush1c* mRNAs with RNAscope (red) (scale bar: 100 µm). (*C*) Z-projection of P8 cochlea cryosections focusing on the scala media compartment and stained for DAPI (gray), immunostained for myosin7a (green), and stained for *Otoa*, *SMPX, Epyc* and *Aqp4* mRNAs with RNAscope (red) (same scale as for (B)). (D) T-SNE plots of scRNAseq data (P8-P12-P20) for the supporting cells showing the levels of *Otoa*, *SMPX, Epyc* and *Aqp4* transcripts.

Figure S6

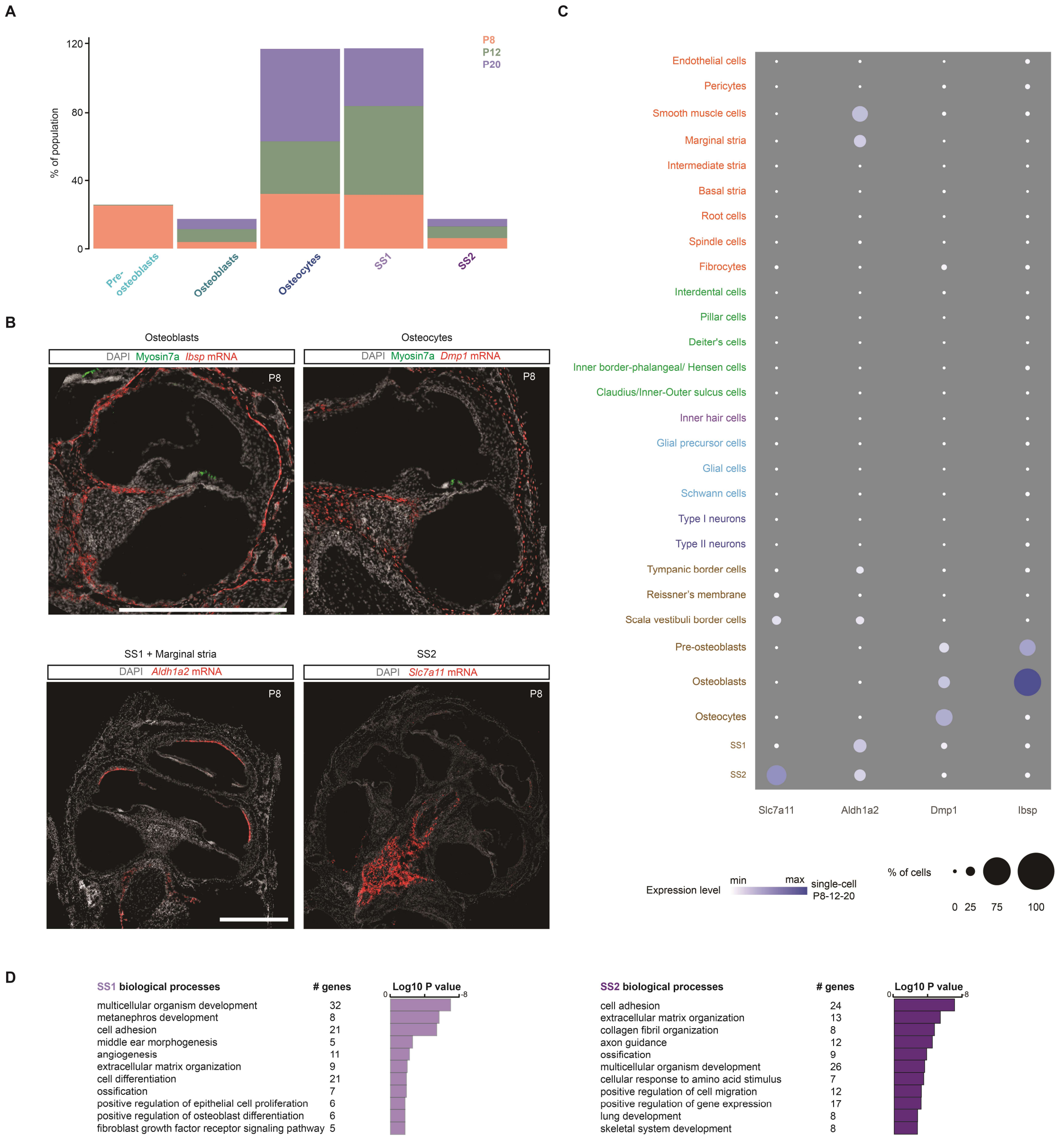

Fig. S6. Transcriptomic identification of the osseous cell types. (*A*) Bar plots displaying the age-related proportions of the osseous cell types. (*B*) Z-projection of P8 cochlea cryosections stained with DAPI (gray), immunostained for myosin7a (green), and stained for *Ibsp* mRNA, a marker of osteoblasts, *Dmp1* mRNA, a marker of osteocytes, and *Aldh1a2* mRNA as well as *Slc7a11* mRNA, markers of SS1 and SS2, respectively, with RNAscope (red) (scale bar: 500 µm). (*C*) Bubble-plot analysis of the scRNAseq dataset (blue, P8-P12-P20). The intensity of the color indicates the level of gene expression, and bubble size indicates the proportion of cells expressing the gene. (*D*) GO term analysis for biological processes in SS1 (left) and SS2 (right), ranked by their statistical significance (Log_{10} *p*- value). The numbers of genes involved in the biological processes are shown.

Figure S7

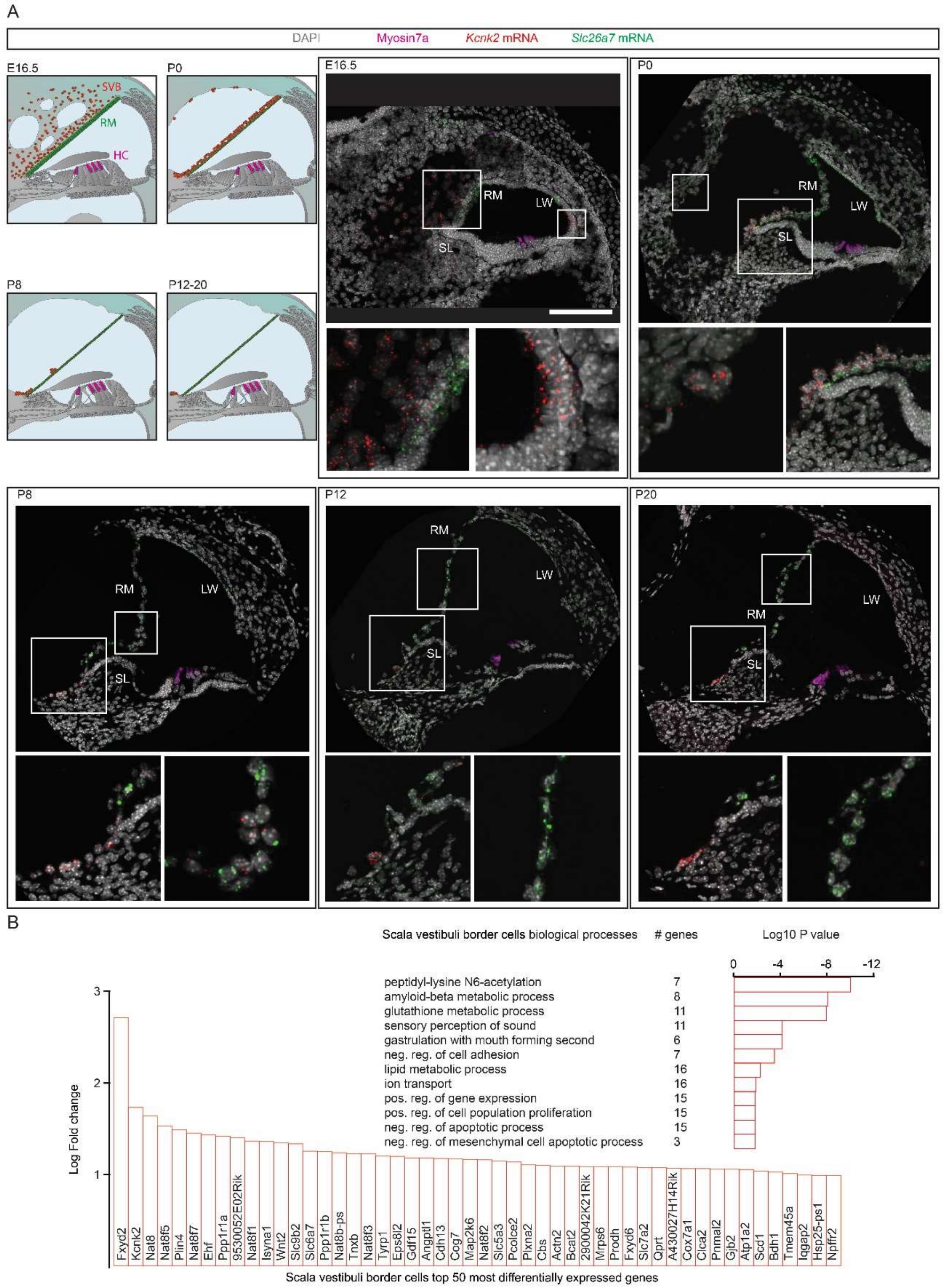

Fig. S7. Developmental characterization of the scala vestibuli border cells (SVBs). (*A*) Diagram of the cochlea at E16.5, P0, P8 and P12-20, highlighting the scala vestibuli border cells (SVB), Reissner's membrane (RM) and the hair cells (HC). Z-projections of E16.5, P0, P8, P12 and P20 cochlear cryosections stained with DAPI (gray), immunostained for myosin7a (magenta), and stained for *Kcnk2* mRNA (red) and *Slc26a7* mRNA (green) with RNAscope. Magnifications of the areas framed in white are shown. Scale bar: 100 µm. (*B*) 50 most differentially expressed genes in the scala vestibuli border cells, ranked by log fold-change in expression relative to other cochlear cell types. Top right: GO-term analysis for the biological processes of the scala vestibuli border cells ranked by statistical significance (log_{10} *p*-value). The numbers of genes involved in the biological processes are shown. SL: Spiral limbus; RM: Reissner's membrane; LW: lateral wall.

Figure S8

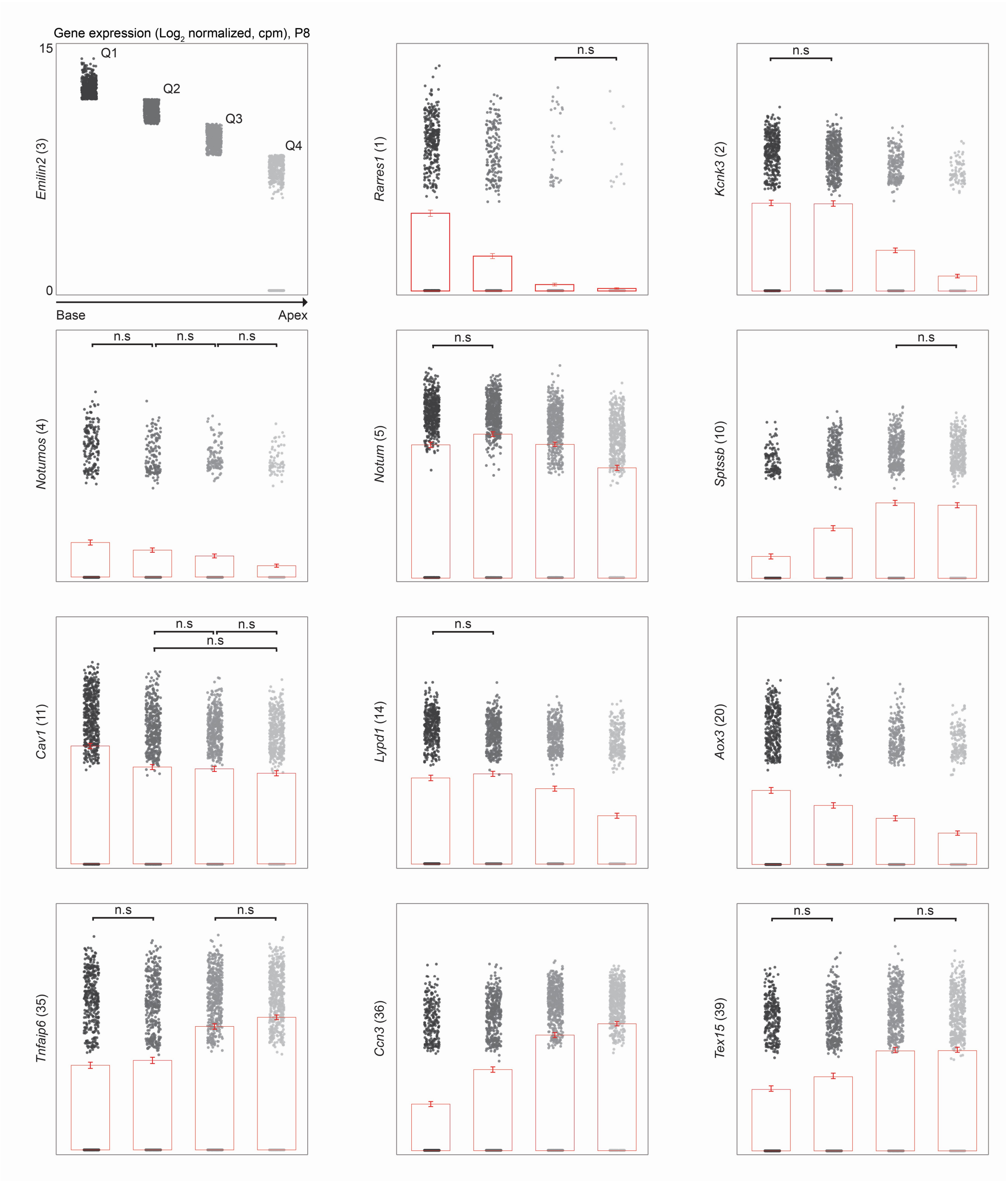

Fig. S8. Tonotopic dynamics of tympanic border cell gene expression. Scatter plots and bar plots (mean + s.e.m) showing the levels of expression on P8 (counts per million, log_2-normalized) of genes differentially expressed in tympanic border cells (ranked among the most differentially expressed genes, indicated in brackets after the gene name of interest) classified according to *Emilin2* expression that is split into four equal quartiles (Q1 to Q4) assumed to represent four tonotopic subregions of the cochlea from the base to the apex, based on RNAscope assays. Non-siginificant differences are indicated by n.s, no indications mean a significant difference. Kruskal-Wallis test followed by NPMC DunnHolland-Wolfe test.

Figure S9-1

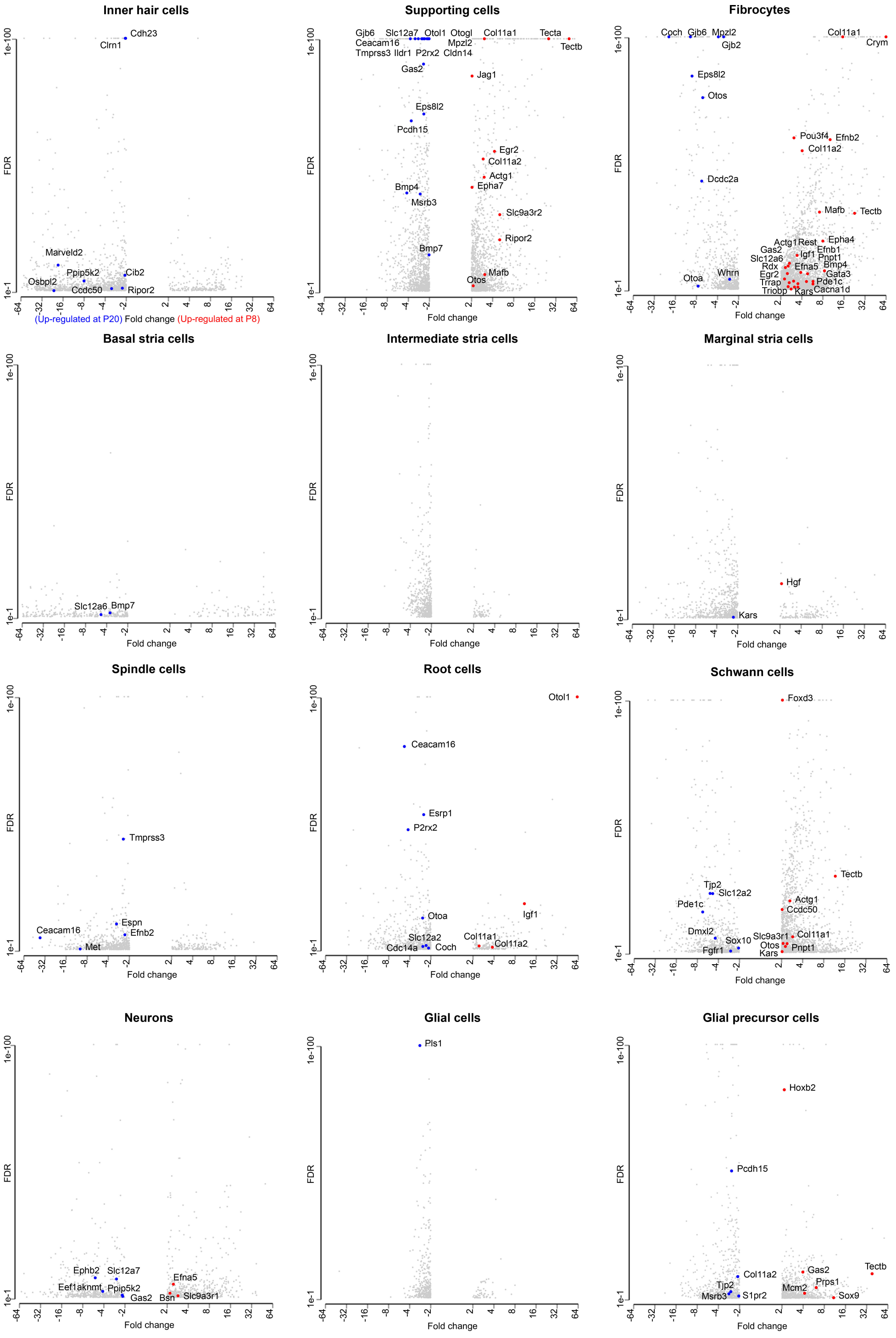

Inner hair cells
Cdh23
Clrn1
Marveld2
Ppip5k2
Cib2
Osbpl2
Ccdc50
Ripor2
(Up-regulated at P20) Fold change (Up-regulated at P8)
Supporting cells
Gjb6
Slc12a7
Otol1
Otogl
Col11a1
Tecta
Ceacam16
Mpzl2
Tectb
Tmprss3
Ildr1
P2rx2
Cldn14
Gas2
Jag1
Eps8l2
Pcdh15
Egr2
Col11a2
Actg1
Bmp4
Epha7
Msrb3
Slc9a3r2
Ripor2
Bmp7
Mafb
Otos
Fibrocytes
Coch
Gjb6
Mpzl2
Col11a1
Gjb2
Crym
Eps8l2
Otos
Pou3f4
Efnb2
Col11a2
Dcdc2a
Mafb
Tectb
Actg1
Rest
Epha4
Gas2
Efnb1
Slc12a6
Igf1
Pnpt1
Rdx
Efna5
Bmp4
Egr2
Gata3
Otoa
Whrn
Trrap
Pde1c
Triobp
Kars
Cacna1d
Basal stria cells
Slc12a6
Bmp7
Intermediate stria cells
Marginal stria cells
Hgf
Kars
Spindle cells
Tmprss3
Ceacam16
Espn
Efnb2
Met
Root cells
Otol1
Ceacam16
Esrp1
P2rx2
Otoa
Igf1
Slc12a2
Col11a1
Cdc14a
Coch
Col11a2
Schwann cells
Foxd3
Tectb
Tjp2
Slc12a2
Pde1c
Actg1
Ccdc50
Dmxl2
Sox10
Slc9a3r1
Col11a1
Fgfr1
Otos
Pnpt1
Kars
Neurons
Ephb2
Slc12a7
Efna5
Eef1aknmt
Ppip5k2
Gas2
Bsn
Slc9a3r1
Glial cells
Pls1
Glial precursor cells
Hoxb2
Pcdh15
Col11a2
Gas2
Tectb
Tjp2
Prps1
Mcm2
Msrb3
S1pr2
Sox9
FDR
1e-100
1e-1
Fold change
-64
-32
-16
-8
-4
-2
2
4
8
16
32
64

Figure S9-2

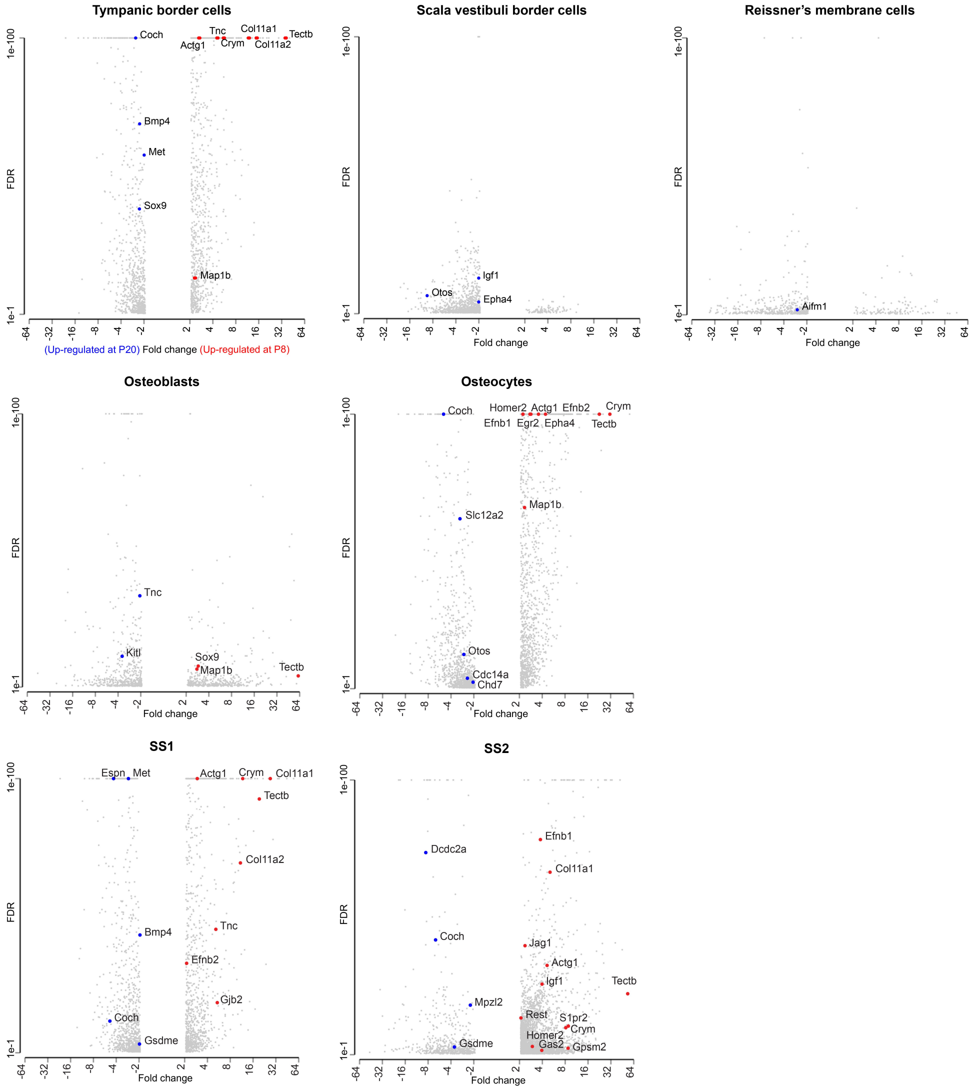

Fig. S9. Differential gene expression during cochlear developmental maturation. Volcano plots displaying the differential pattern of gene expression between P8 and P20 for the various cochlear cell types. Fold-changes in expression are indicated on the *x* axis, with positive values indicating an upregulation on P8 and negative values indicating an upregulation on P20. The false-discovery rate-corrected *p*-value is indicated on the *y* axis. Only genes with a fold-change in expression of at least 2 in either direction (-2/+2) and an FDR-corrected *p*-value less than 0.05 are displayed in gray. The upregulated deafness/key genes are highlighted in red or blue for P8 and P20, respectively.

Figure S10-1

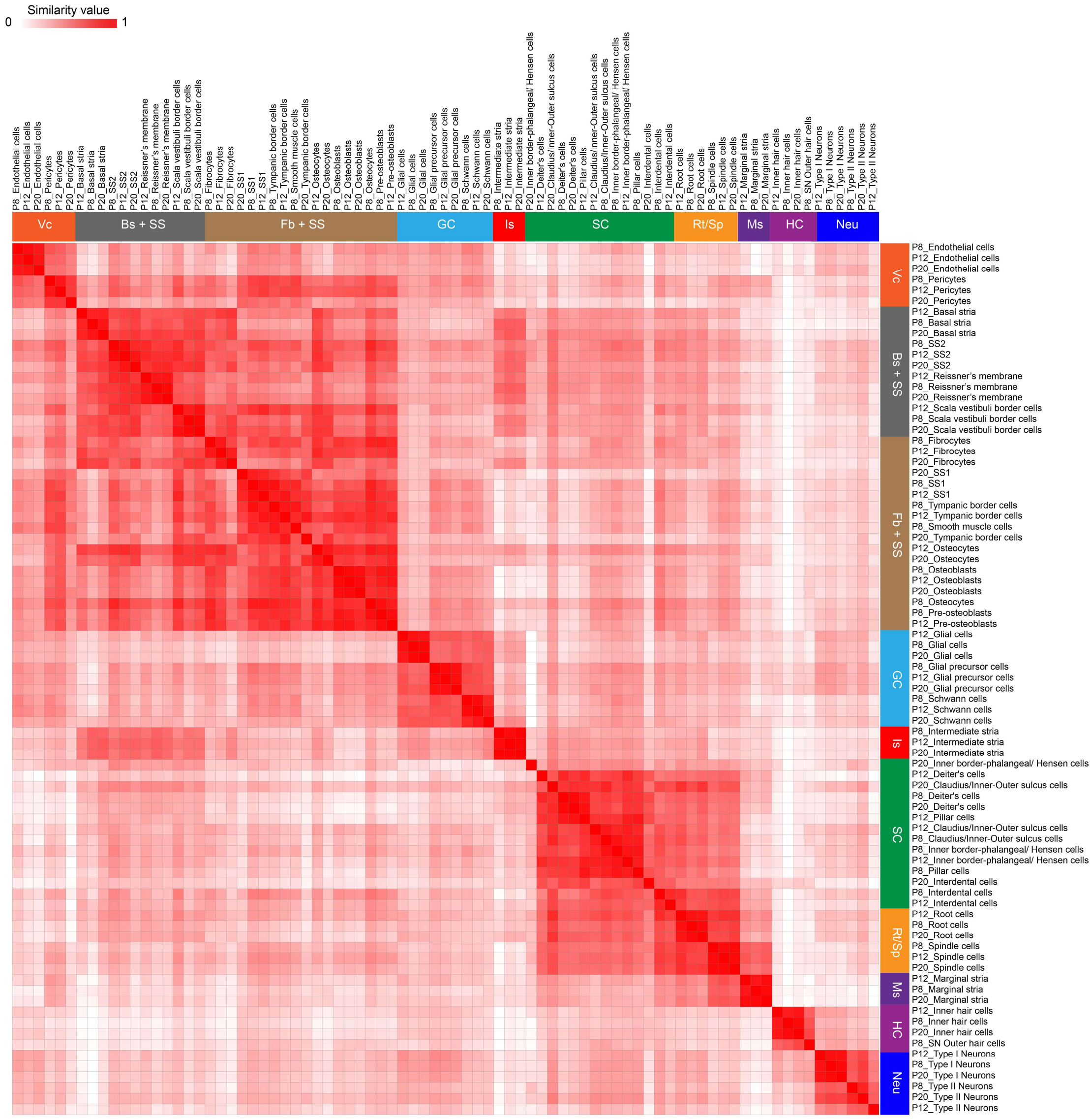
Similarity value
0
1
Vc
Bs + SS
Fb + SS
GC
Is
SC
Rt/Sp
Ms
HC
Neu
P8_Endothelial cells
P12_Endothelial cells
P20_Endothelial cells
P8_Pericytes
P12_Pericytes
P20_Pericytes
P12_Basal stria
P8_Basal stria
P20_Basal stria
P8_SS2
P12_SS2
P20_SS2
P12_Reissner's membrane
P8_Reissner's membrane
P20_Reissner's membrane
P12_Scala vestibuli border cells
P8_Scala vestibuli border cells
P20_Scala vestibuli border cells
P8_Fibrocytes
P12_Fibrocytes
P20_Fibrocytes
P20_SS1
P8_SS1
P12_SS1
P8_Tympanic border cells
P12_Tympanic border cells
P8_Smooth muscle cells
P20_Tympanic border cells
P12_Osteocytes
P20_Osteocytes
P8_Osteoblasts
P12_Osteoblasts
P20_Osteoblasts
P8_Osteocytes
P8_Pre-osteoblasts
P12_Pre-osteoblasts
P12_Glial cells
P8_Glial cells
P20_Glial cells
P8_Glial precursor cells
P12_Glial precursor cells
P20_Glial precursor cells
P8_Schwann cells
P12_Schwann cells
P20_Schwann cells
P8_Intermediate stria
P12_Intermediate stria
P20_Intermediate stria
P20_Inner border-phalangeal/ Hensen cells
P12_Deiter's cells
P20_Claudius/Inner-Outer sulcus cells
P8_Deiter's cells
P20_Deiter's cells
P12_Pillar cells
P12_Claudius/Inner-Outer sulcus cells
P8_Claudius/Inner-Outer sulcus cells
P8_Inner border-phalangeal/ Hensen cells
P12_Inner border-phalangeal/ Hensen cells
P8_Pillar cells
P20_Interdental cells
P8_Interdental cells
P12_Interdental cells
P12_Root cells
P8_Root cells
P20_Root cells
P8_Spindle cells
P12_Spindle cells
P20_Spindle cells
P12_Marginal stria
P8_Marginal stria
P20_Marginal stria
P12_Inner hair cells
P8_Inner hair cells
P20_Inner hair cells
P8_SN Outer hair cells
P12_Type I Neurons
P8_Type I Neurons
P20_Type I Neurons
P8_Type II Neurons
P20_Type II Neurons
P12_Type II Neurons

Figure S10-2

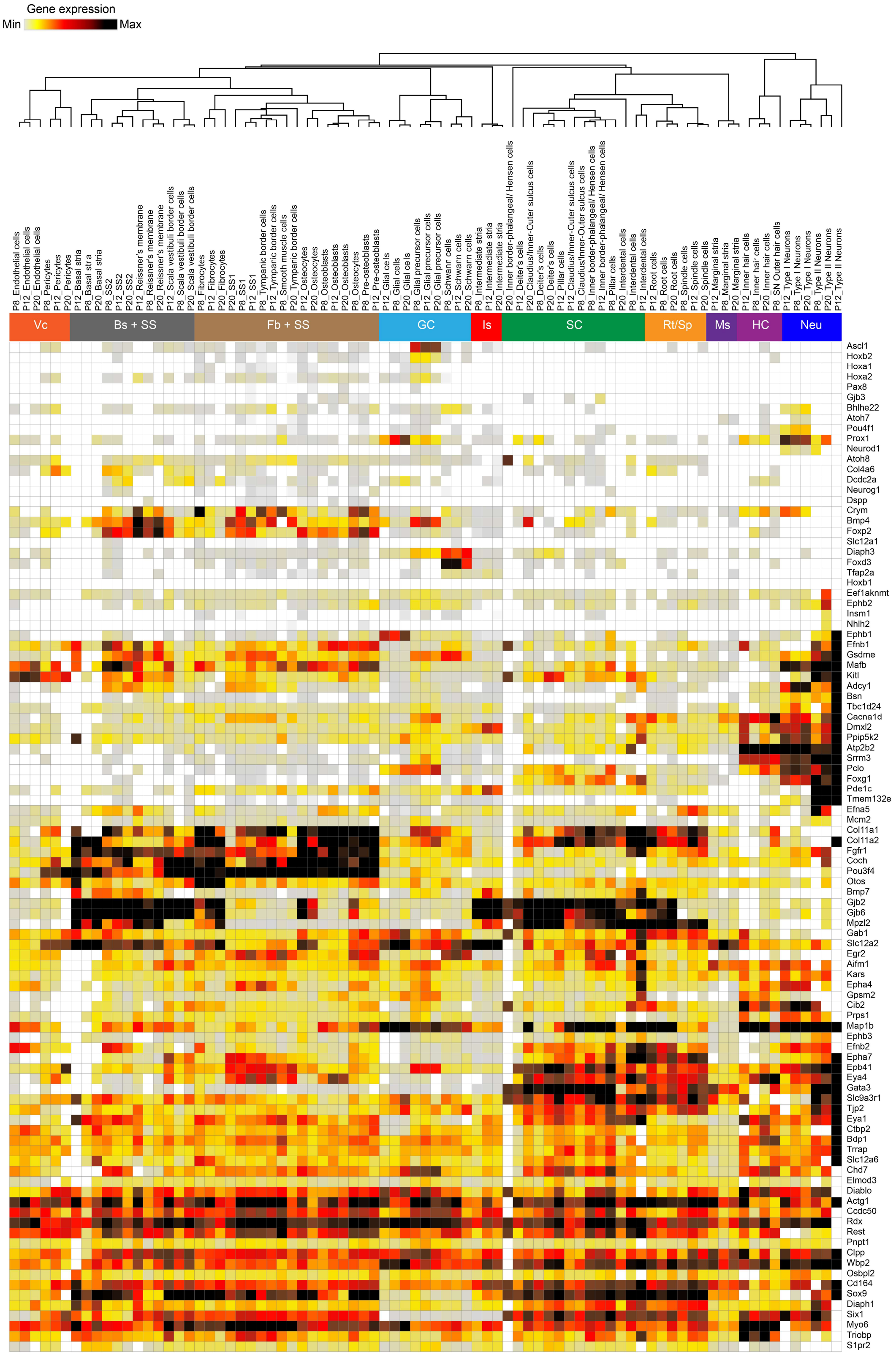

Gene expression
Min
Max
P8_Endothelial cells
P12_Endothelial cells
P20_Endothelial cells
P8_Pericytes
P12_Pericytes
P20_Pericytes
P12_Basal stria
P8_Basal stria
P20_Basal stria
P8_SS2
P12_SS2
P20_SS2
P12_Reissner's membrane
P8_Reissner's membrane
P20_Reissner's membrane
P12_Scala vestibuli border cells
P8_Scala vestibuli border cells
P20_Scala vestibuli border cells
P8_Fibrocytes
P12_Fibrocytes
P20_Fibrocytes
P20_SS1
P8_SS1
P12_SS1
P8_Tympanic border cells
P12_Tympanic border cells
P8_Smooth muscle cells
P20_Tympanic border cells
P12_Osteocytes
P20_Osteocytes
P8_Osteoblasts
P12_Osteoblasts
P20_Osteoblasts
P8_Osteocytes
P8_Pre-osteoblasts
P12_Pre-osteoblasts
P12_Glial cells
P8_Glial cells
P20_Glial cells
P8_Glial precursor cells
P12_Glial precursor cells
P8_Schwann cells
P12_Schwann cells
P20_Schwann cells
P8_Intermediate stria
P12_Intermediate stria
P20_Intermediate stria
P20_Inner border-phalangeal/ Hensen cells
P12_Deiter's cells
P20_Claudius/Inner-Outer sulcus cells
P8_Deiter's cells
P20_Deiter's cells
P12_Pillar cells
P12_Claudius/Inner-Outer sulcus cells
P8_Claudius/Inner-Outer sulcus cells
P8_Inner border-phalangeal/ Hensen cells
P12_Inner border-phalangeal/ Hensen cells
P8_Pillar cells
P20_Interdental cells
P8_Interdental cells
P12_Interdental cells
P12_Root cells
P8_Root cells
P20_Root cells
P8_Spindle cells
P12_Spindle cells
P20_Spindle cells
P12_Marginal stria
P8_Marginal stria
P20_Marginal stria
P12_Inner hair cells
P8_Inner hair cells
P20_Inner hair cells
P8_SN Outer hair cells
P12_Type I Neurons
P8_Type I Neurons
P20_Type I Neurons
P8_Type II Neurons
P20_Type II Neurons
P12_Type II Neurons
Vc
Bs + SS
Fb + SS
GC
Is
SC
Rt/Sp
Ms
HC
Neu
Ascl1
Hoxb2
Hoxa1
Hoxa2
Pax8
Gjb3
Bhlhe22
Atoh7
Pou4f1
Prox1
Neurod1
Atoh8
Col4a6
Dcdc2a
Neurog1
Dspp
Crym
Bmp4
Foxp2
Slc12a1
Diaph3
Foxd3
Tfap2a
Hoxb1
Eef1aknmt
Ephb2
Insm1
Nhlh2
Ephb1
Efnb1
Gsdme
Mafb
Kitl
Adcy1
Bsn
Tbc1d24
Cacna1d
Dmxl2
Ppip5k2
Atp2b2
Srrm3
Pclo
Foxg1
Pde1c
Tmem132e
Efna5
Mcm2
Col11a1
Col11a2
Fgfr1
Coch
Pou3f4
Otos
Bmp7
Gjb2
Gjb6
Mpzl2
Gab1
Slc12a2
Egr2
Aifm1
Kars
Epha4
Gpsm2
Cib2
Prps1
Map1b
Ephb3
Efnb2
Epha7
Epb41
Eya4
Gata3
Slc9a3r1
Tjp2
Eya1
Ctbp2
Bdp1
Trrap
Slc12a6
Chd7
Elmod3
Diablo
Actg1
Ccdc50
Rdx
Rest
Pnpt1
Clpp
Wbp2
Osbpl2
Cd164
Sox9
Diaph1
Six1
Myo6
Triobp
S1pr2

Figure S10-3

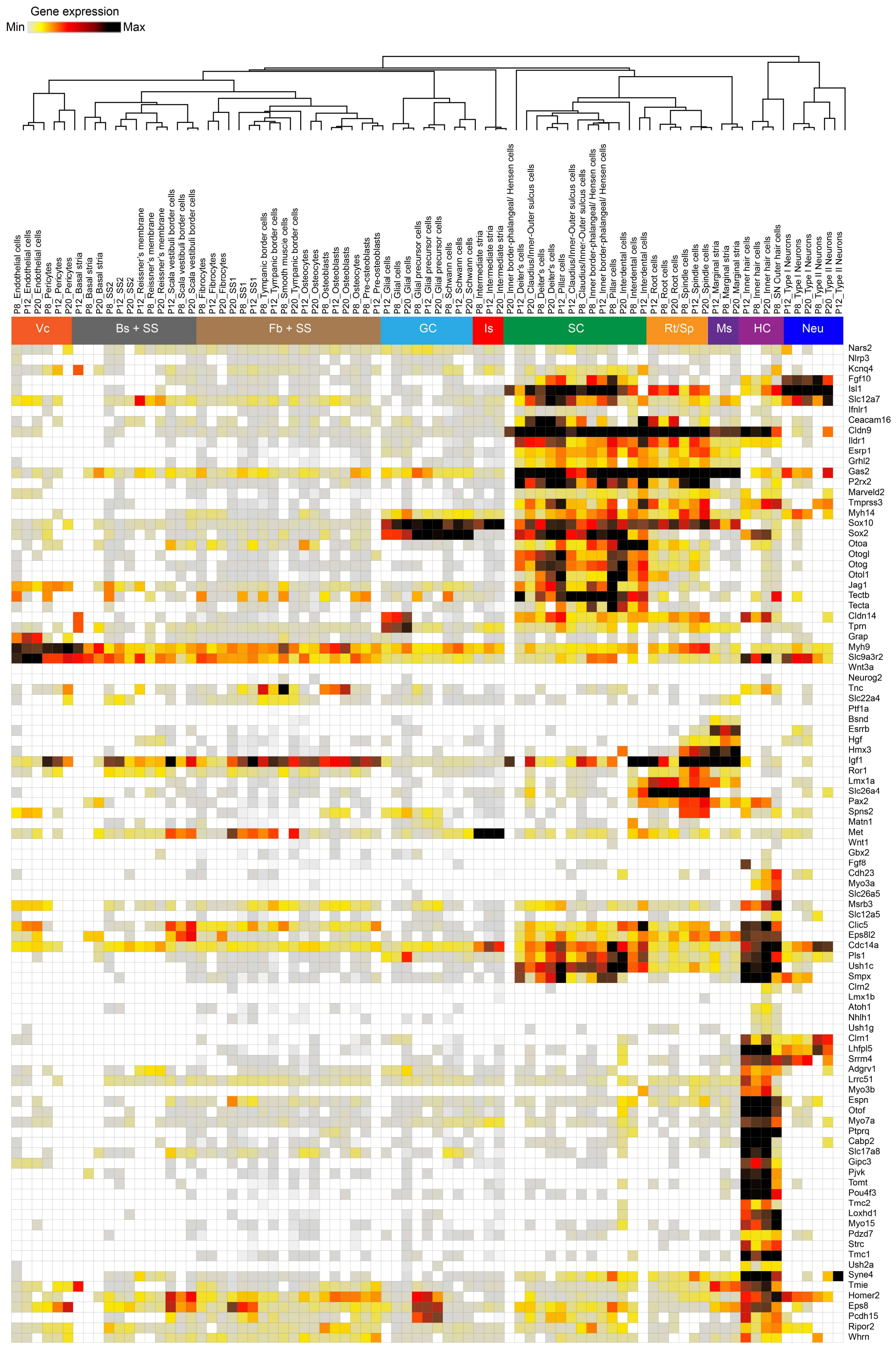

Gene expression
Min
Max
P8_Endothelial cells
P12_Endothelial cells
P20_Endothelial cells
P8_Pericytes
P12_Pericytes
P20_Pericytes
P12_Basal stria
P8_Basal stria
P20_Basal stria
P8_SS2
P12_SS2
P20_SS2
P12_Reissner's membrane
P8_Reissner's membrane
P20_Reissner's membrane
P12_Scala vestibuli border cells
P8_Scala vestibuli border cells
P20_Scala vestibuli border cells
P8_Fibrocytes
P12_Fibrocytes
P20_Fibrocytes
P20_SS1
P8_SS1
P12_SS1
P8_Tympanic border cells
P12_Tympanic border cells
P8_Smooth muscle cells
P20_Tympanic border cells
P12_Osteocytes
P20_Osteocytes
P8_Osteoblasts
P12_Osteoblasts
P20_Osteoblasts
P8_Osteocytes
P8_Pre-osteoblasts
P12_Pre-osteoblasts
P12_Glial cells
P8_Glial cells
P20_Glial cells
P8_Glial precursor cells
P12_Glial precursor cells
P20_Glial precursor cells
P8_Schwann cells
P12_Schwann cells
P20_Schwann cells
P8_Intermediate stria
P12_Intermediate stria
P20_Intermediate stria
P20_Inner border-phalangeal/ Hensen cells
P12_Deiter's cells
P20_Claudius/Inner-Outer sulcus cells
P8_Deiter's cells
P20_Deiter's cells
P12_Pillar cells
P12_Claudius/Inner-Outer sulcus cells
P8_Claudius/Inner-Outer sulcus cells
P8_Inner border-phalangeal/ Hensen cells
P12_Inner border-phalangeal/ Hensen cells
P8_Pillar cells
P20_Interdental cells
P8_Interdental cells
P12_Interdental cells
P12_Root cells
P8_Root cells
P20_Root cells
P8_Spindle cells
P12_Spindle cells
P20_Spindle cells
P12_Marginal stria
P8_Marginal stria
P20_Marginal stria
P12_Inner hair cells
P8_Inner hair cells
P20_Inner hair cells
P8_SN Outer hair cells
P12_Type I Neurons
P8_Type I Neurons
P20_Type I Neurons
P8_Type II Neurons
P20_Type II Neurons
P12_Type II Neurons
Vc
Bs + SS
Fb + SS
GC
Is
SC
Rt/Sp
Ms
HC
Neu
Nars2
Nlrp3
Kcnq4
Fgf10
Isl1
Slc12a7
Ifnlr1
Ceacam16
Cldn9
Ildr1
Esrp1
Grhl2
Gas2
P2rx2
Marveld2
Tmprss3
Myh14
Sox10
Sox2
Otoa
Otogl
Otog
Otol1
Jag1
Tectb
Tecta
Cldn14
Tprn
Grap
Myh9
Slc9a3r2
Wnt3a
Neurog2
Tnc
Slc22a4
Ptf1a
Bsnd
Esrrb
Hgf
Hmx3
Igf1
Ror1
Lmx1a
Slc26a4
Pax2
Spns2
Matn1
Met
Wnt1
Gbx2
Fgf8
Cdh23
Myo3a
Slc26a5
Msrb3
Slc12a5
Clic5
Eps8l2
Cdc14a
Pls1
Ush1c
Smpx
Clrn2
Lmx1b
Atoh1
Nhlh1
Ush1g
Clrn1
Lhfpl5
Srrm4
Adgrv1
Lrrc51
Myo3b
Espn
Otof
Myo7a
Ptprq
Cabp2
Slc17a8
Gipc3
Pjvk
Tomt
Pou4f3
Tmc2
Loxhd1
Myo15
Pdzd7
Strc
Tmc1
Ush2a
Syne4
Tmie
Homer2
Eps8
Pcdh15
Ripor2
Whrn

Fig. S10. Expression patterns for non-syndromic deafness genes in cochlear cells. (*1*) Similarity matrix built on the hierarchical clustering of cochlear cell types based on the expression of 195 deafness genes and key regulatory genes, for the scRNAseq (P8, P12, P20) and snRNAseq (P8, OHCs only) data. The *x* and *y* axes depict the cell types, and the heat map indicates the similarity, with white and red for low and high values, respectively. (*2-3)* Hierarchical clustering of cochlear cell types (*x* axis) and 195 deafness/key genes (*y* axis). The heat map indicates the level of gene expression, with white/yellow and red/black for low and high values, respectively. Vc: vascular cells (endothelial cells, pericytes and smooth muscle cells), Bs + SS: basal stria cells + surrounding structures, Fb + SS: fibrocytes + surrounding structures, GC: glial cells, Is: intermediate stria cells, SC: supporting cells, Rt/Sp: root cells and spindle cells, Ms: marginal stria cells, HC: hair cells, Neu: neurons.

DATASET LEGENDS

Dataset S1. Differential gene expression ranking of cochlear cell types. The differentially expressed genes in each cochlear cell type, separated by age and sequencing techniques, are ranked by *p*-values. The cell types are gathered by cell ensembles. Detected deafness genes are indicated by "^", detected key genes regulating cochlear development and function are indicated by "*".

Dataset S2. Gene Ontology term analysis of cochlear cell types. For every cell types, all ages combined (P8-P12-P20), taking into account the 200 most differentially expressed genes; the gene ontology (GO) terms are indicated by their reference number (# GO), with the name of the associated biological process (name), number of genes implicated in this process among the 200 investigated (ratio_matching_genes_in_current_study), the number of genes involved in these processes among the whole genome (ratio_matching_genes_in_whole_gene_pop), the depth of the process (GO_term_depth_level), the corrected *p*-value (FDR), and the gene names detailed (matching_genes_in_current_study).

Dataset S3. Differential gene expression ranking in tympanic border cells with a tonotopic gradient. The differentially expressed genes are ranked by *p*-values. The average gene expression (counts per million, log_2-normalized) is indicated for the four quartiles (605 cells each) corresponding to each tonotopic subregion. The ratio of the expression base/apex or apex/base as an indication of tonotopic gradient strength is shown. The ones decreasing or increasing from base to apex are colored in blue and red, respectively. The full gene names are indicated for the genes mentioned in the main text. The transcription factors are indicated by "&".

Dataset S4. Differential gene expression between P8 and P20. For every cell type, the genes differentially expressed between P8 and P20 (P8 vs P20) exhibiting a corrected *p*-value (FDR) inferior or equal than 0.5, and a fold change of expression inferior to -2 or superior to +2 are shown. Negative and positive fold changes indicate upregulation at P20 and P8, respectively. The percentage of cells within the cell type, expressing the given gene at each stage are shown. The transcription factors, deafness genes and deafness/key genes are indicated by "&", "^" and "@", respectively.

Dataset S5. Probes used for RNAscope assays. All the RNAscope probes used in the study are indicated, with their reference, targeted transcripts, product number and optionally the figure where their associated stainings appear.

SI REFERENCES

1. N. F. Fernandez, *et al.*, Clustergrammer, a web-based heatmap visualization and analysis tool for high-dimensional biological data. *Sci Data* **4**, 170151 (2017).
2. D. V. Klopfenstein, *et al.*, GOATOOLS: A Python library for Gene Ontology analyses. *Sci Rep* **8**, 10872 (2018).
3. H. Mi, A. Muruganujan, D. Ebert, X. Huang, P. D. Thomas, PANTHER version 14: more genomes, a new PANTHER GO-slim and improvements in enrichment analysis tools. *Nucleic Acids Res* **47**, D419–D426 (2019).
4. P. Shannon, *et al.*, Cytoscape: A Software Environment for Integrated Models of Biomolecular Interaction Networks. *Genome Res.* **13**, 2498–2504 (2003).
